# Short-Chained Platinum Complex Catalyzed Hydrosilylation under Thermomorphic Conditions: Heterogeneous Phase Separation at Ice Temperature

**DOI:** 10.3390/molecules26020378

**Published:** 2021-01-13

**Authors:** Chiao-Fan Chiu, Jinn-Hsuan Ho, Eskedar Tessema, Yijing Lu, Chia-Rui Shen, Chang-Wei Lin, Norman Lu

**Affiliations:** 1 Department of Pediatrics, Linkou Medical Center, Chang Gung Memorial Hospital, Taoyuan 333, Taiwan; chioufan2008@gmail.com; 2 Graduate Institute of Clinical Medical Sciences, College of Medicine, Chang Gung University, Taoyuan 333, Taiwan; 3 Department of Chemical Engineering, National Taiwan University of Technology, Taipei 106, Taiwan; jhho@mail.ntust.edu.tw; 4 Institute of Organic and Polymeric Materials, National Taipei University of Technology, Taipei 106, Taiwan; eskedartessema18@gmail.com (E.T.); yijinglu@hotmail.com.tw (Y.L.); 5 Department of Medical Biotechnology and Laboratory Sciences, College of Medicine, Chang Gung University, Taoyuan 333, Taiwan; crshen@mail.cgu.edu.tw; 6 Department of Ophthalmology, Linkou Medical Center, Chang Gung Memorial Hospital, Taoyuan 333, Taiwan; 7 Department of Internal Medicine, Division of Pulmonary and Critical Care Medicine, Chang Gung Memorial Hospital & Chang Gung University, Taoyuan 333, Taiwan; 8 Development Center for Smart Textile, National Taipei University of Technology, Taipei 106, Taiwan

**Keywords:** heterogeneous separation, catalyst, fluorous, hydrosilylation, platinum, thermomorphic, recovery, selectivity

## Abstract

Homogeneous catalysts PtCl_2_[5,5′-bis-(*n*-ClCF_2_(CF_2_)_3_CH_2_OCH_2_)-2,2′-bpy] (**2A**) and PtCl_2_[5,5′-bis-(*n*-HCF_2_(CF_2_)_3_CH_2_OCH_2_)-2,2′-bpy] (**2B**), which contained short fluorous chains, were synthesized and used in catalysis of hydrosilylation of alkynes. In these reactions the thermomorphic mode was effectively used to recover these catalysts from the reaction mixture up to eight cycles by taking advantage of heterogeneous phase separation at ice temperature. This kind of catalysis had previously been observed in fluorous catalysts of platinum containing about 50% F-content, but in this work the percentage of F-content is decreased to only about 30%, by which we termed them as “very light fluorous”. Our new type of catalyst with limited number of F-content is considered as the important discovery in the fluorous technology field as the reduced number of fluorine atoms will help to be able to comply the EPA 8-carbon rule. The metal leaching after the reaction has been examined by ICP-MS, and the testing results show the leaching of residual metal to be minimal. Additionally, comparing these results to our previous work, fluorous chain assisted selectivity has been observed when different fluorous chain lengths of the catalysts are used. It has been found that there exists fluorous chain assisted better selectivity towards β-(*E*) form in the Pt-catalyzed hydrosilylation of non-symmetric terminal alkyne when the Pt catalyst contains short fluorous chain (i.e., 4 Cs). Phenyl acetylenes showed the opposite regioselectivity due to pi-pi interaction while using the same catalyst via Markovnikov’s addition to form terminal vinyl silane, which is then a major product for Pt-catalyzed hydrosilylation of terminal aryl acetylene with triethylsilane. Finally, the kinetic studies indicate that the insertion of alkyne into the Pt-H bond is the rate-determining step.

## 1. Introduction

Developing recyclable catalysts, able to reduce the waste of expensive transition metals is one of the important issues in sustainable research. Generally, homogeneous catalysts have good catalytic activity but they have limitation on separation catalytic recovery [1], whereas heterogeneous catalysts are easily recyclable but with low catalytic activity [2,3,4]. A valuable method, called thermomorphic system provides a good resolution which makes the catalyst dissolve in one condition but precipitate in another. Using the special solubility selectivity of fluorous chain-containing metal complex [5,6] is very promising for the catalytic thermomorphic system in the development of fluorine chemistry [7].

Many polyfluorinated chain-containing metal complexes are used in the fluorous biphasic system (FBS) [8] which is an immiscible two-solvent system having one fluorous solvent and a common organic one. After reaction completion, the fluorous chain-containing metal complexes can be easily extracted from the fluorous solvent while the other organic products stay in organic layer. To ensure the good solubility for fluorous chain-containing compounds soluble in fluorous solvents, “heavy fluorous content” [9] (more than 60% fluorine content, F-content) is required. This may result in a big drawback [10]; that is the need for expensive fluorous starting material and the solvent. To reduce the F-content of compounds and fluorous solvent, an alternative way is to use the different solubility of “light fluorous compounds” (less than 60% of F-content) [5,11] between high and low temperature in a common organic solvent. In this method, a fluorous chain-containing metal catalyst could be soluble at high temperature during the catalytic reactions, but insoluble at room temperature (or lower temperature) for the recovery of catalysts via a simple liquid-solid separation [12,13,14,15,16,17]. This is a typical application of “light fluorous” metal catalysts in the so-called “thermomorphic condition”.

A variety of fluorous chain-containing metal complexes have been synthesized in our laboratory and studied for the possible applications [18,19]. In 2016, we have demonstrated the first example of using a fluorous chain-containing Pt complex with heavy fluorine content {[PtCl_2_[5,5′-bis-(*n*-C_11_F_23_CH_2_OCH_2_)-2,2′-bpy] (**3a**), with 53% F-content for the Pt catalyst} successfully catalyzed the hydrosilylation of alkynes in dibutyl ether (DBE) under thermomorphic condition [19]. The results of the catalytic hydrosilylation reaction showed high reaction yields, good turnover numbers and good recovery of the catalyst for up to 8 times of re-usage with very low leaching level of Pt (ca. 0.001%).

The hydrosilylation of alkynes has been performed with a variety of metal catalysts, including Pt, Ir, Ru, Rh, and Co complexes, and usually produced three possible products, α, β-(*E*) and β-(*Z*) isomers [20,21,22,23,24,25,26,27,28,29]. Generally, β-(*E*) is the thermo-dynamically favored isomer and dominates in most researches using aliphatic and aryl alkynes, such as Lewis’s work using Karstedt’s Pt catalyst [30], Marko’s work using *N-*heterocyclic carbene Pt(0) catalysts (>95% yield) [31], Ge’s work using bisphosphine Co catalysts (60–90% yield) [32], Jesus’s work using water-soluble *N*-heterocyclic carbene Pt(0) Complexes as a recyclable catalyst (58–97% yield) [33,34], and Mata’s work using a recoverable Pt catalyst immobilized on the surface of graphene (80–99% yield) [35]. Additionally, Chauhan and Sarkar’s work using Pt-nanoparticle catalyst (95–98% yield) [36] and Cai’s work using a reusable K_2_PtCl_4_/Xphos-SO_3_Na/PEG-400/H_2_O system (72–93% yield) [37] were reported to be efficient and recyclable to give β-(*E*) vinylsilane. However, α isomer has also been achieved to be the major product in few studies, such as Corma’s work, which only presented for aryl acetylenes, using ligand-free Pt_3_ cluster catalyst (65–93% yield) [38], Trost’s work using pentamethylcyclopentadiene Ru catalysts (61–92% yield) [39], and Huang’s work using iminopyridine-oxazoline Co catalyst (55–95% yield) [40]. Additionally, Mori’s work reported RhI-based catalyst which gave the β-(*Z*) isomer as a dominant product for both aliphatic and aryl alkynes [41]. This study, which is focused on recoverable catalysis, successfully developed a new recyclable Pt catalyst **2A** under thermomorphic condition to afford only two isomeric products, α and β-(*E*) isomers, with better yield (>99%), where the major products usually being β-(*E*) isomer for the aliphatic alkynes and α-vinylsilanes for the aryl alkynes in the hydrosilylation of terminal alkynes. All the results with usual features are discussed below.

It is important [42] that we could further lower the F-content of catalyst, in a way that its F-content is even lower than that of so-called “light fluorous” catalyst; and is without compromising the separation effectiveness, so they become readily adapted to the conventional chemical and biochemical processes. In this work, the even shorter fluorous chain-containing platinum catalysts, [PtCl_2_(5,5′-bis(CH_2_OCH_2_C_4_F_8_Cl)-2,2′-bpy)] (**2A**) and [PtCl_2_(5,5′-bis(CH_2_OCH_2_C_4_F_8_H)-2,2′-bpy)] (**2B**) with the even lower fluorine content (i.e., 31.0 and 33.4% F-content for **2A** and **2B,** respectively.) have been synthesized and been applied to catalyze the hydrosilylation reactions of internal and terminal alkynes. To the best of our knowledge, this is the first example of the application of very short polyfluorinated Pt complexes as effective, recoverable hydrosilylation catalysts under thermomorphic condition showing heterogeneous phase separation at ice temperature. These “very light fluorous” Pt complexes **2A** and **2B** feature on homogeneously catalyzing reaction at high temperature in common organic solvent and turning to the heterogeneous precipitation at lower temperature under thermomorphic condition. Taking into consideration of their good catalytic capabilities and good thermal stability, these catalysts could be regarded as the very good alternatives to most of the homogeneous catalysts.

## 2. Results and Discussion

### 2.1. Catalyst Synthesis

**Scheme 1:**
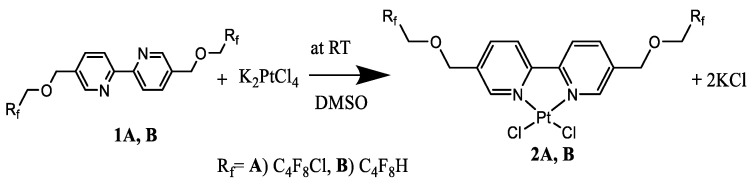
Synthesis of the short fluorous chain Pt complexes.

The preparation of ligands bis-5,5′-(*n*-ClCF_2_(CF_2_)_3_CH_2_OCH_2_)-2,2′-bpy (**1A**) and bis-5,5′-(*n*-HCF_2_(CF_2_)_3_CH_2_OCH_2_)-2,2′-bpy (**1B**) followed a literature procedure [43]. The ligand of **1A** or **1B** has the lower F-content (wt. %), which is 42.6 and 47.2%, respectively. The reaction of fluorinated bipyridine (bpy) ligands, **1A, 1B,** with K_2_PtCl_4_ in DMSO, as shown in Scheme 1, resulted in the synthesis of Pt complexes PtCl_2_[5,5′-bis-(*n*-ClC_4_F_8_CH_2_OCH_2_)-2,2′-bpy] (**2A**) and PtCl_2_[5,5′-bis-(*n*-HC_4_F_8_CH_2_OCH_2_)-2,2′-bpy] (**2B**) as red solids, respectively. Compounds of **2A** and **2A** exhibit the F-content being 31.0 and 33.4%, respectively; and are soluble in Bu_2_O at 120 °C forming a red color solution. However, compounds **2A** and **2B** are insoluble in Bu_2_O at 25 °C (see Figures S3 and S4 & Scheme S1). Complexes of **2A** and **2B** are also abbreviated as **55-8FCl-PtCl_2_** and **55-8FH-PtCl_2_**, respectively.

### 2.2. Recoverable Pt Complex-Catalyzed Hydrosilylation of Alkynes

The fluorinated short-chained Pt complex **2A** and **2B** were then examined for the following experiments to catalyze the addition of HSiEt_3_ (**3**) to (a) symmetrical internal alkyne 5-decyne (**4**); (b) terminal alkynes 1-decyne (**5**), 1-hexyne (**6**) and functionalized terminal propargylic alkyne (**7**); and (c) aromatic terminal alkynes (**8**–**10**) in the following hydrosilylation reactions.

(a) Symmetrical internal alkyne

**Scheme 2:**
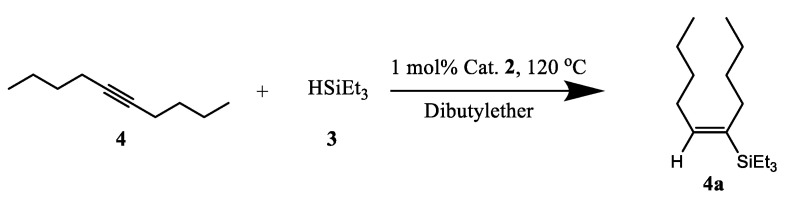
The Pt catalyst (**2A** or **2B**)-catalyzed hydrosilylation of 5-decyne (**4**) with triethylsilane (**3**) under thermomorphic condition.

The Pt catalyst (**2A** or **2B**)-catalyzed hydrosilylation of 5-decyne (**4**) with triethylsilane (**3**) under thermomorphic condition was successfully carried out to afford product **4a** in quantitative conversions, as shown in Scheme 2, whereas no product **4a** could be found in the reaction without using Pt catalyst for 24 h. The best reaction condition for the catalyst **2A** was optimized at 120 °C for 2 h via the GC-MS examination of the reaction while the NMP was used as the internal standard. The quantitative conversion of the hydrosilylations were still observed even over the eight cycles of Pt catalyst recycling almost without decreasing the activity under the thermomorphic condition (Table 1). The similar catalytic effect on the converting yield is also shown by Pt catalyst **2B** whose structure is to have H atom to replace Cl atom of **2A** at the terminal of fluorous chains.

Generally, catalysts with high F-content have good performance under thermomorphic condition, e.g., PtCl_2_[5,5′-bis-(*n*-C_11_F_23_CH_2_OCH_2_)-2,2′-bpy] **(55-23F-PtCl_2_)** reported in our previous work (with C_11_F_23_ moiety, %F = 53) [19], but the catalyst **2A** here only containing “very-light fluorous” chain (e.g., C_4_F_8_, %F = 31) still showed the impressive catalytic activity and efficient recovery under thermomorphic condition (Figures S1 and S3) shown in Table 1.

Generally speaking, typical Pt-based catalysts for the hydrosilylation of alkynes can be used at significantly lower Pt/substrate ratios than 1 mol% loading. We mainly use 1 mol% of catalyst **2A** loading because the scope of this study is on recycling and reuse of catalyst. The hydrosilylation of 5-decyne (**4**) with triethylsilane (**3**) was also reported by F. Alonso’s group using Pt/TiO_2_ as the catalyst by using more stringent catalytic loading of 0.25 mol% [44]. They showed catalytic results under very short reaction time (less than 2 h) at first two catalytic reaction cycles, but the catalyst’s activity was significantly decreased to make the third-cycle reaction taking 3 h for the same conversion and the fifth-cycle reaction giving only 62% conversion after 24 h. In contrast, the fifth-cycle hydrosilylation in our work still had very good performance of 99.5% conversion (entry 5 in Table 1), which indicates that Pt catalyst **2A** is both very efficient and robust. The Pt catalyst **2A**-catalyzed hydrosilylations of 5-decyne (**4**) with triethylsilane (**3**) in Bu_2_O have also been studied kinetically at 120 and 130 °C. These two sets of data points are shown in Section S3 in Supplementary Materials. In both cases, the integrated rate law derived from the alkyne concentration vs time plots is ln[5-decyne] = ln[5-decyne]_0_-kt, with k = 0.069 (R^2^ = 0.95) & 0.099 (R^2^ = 0.99) at 120 °C and 130 °C, respectively. [Note: ln[5-decyne]_0_ = 0]; at 130 °C the derived ln[5-decyne]_0_ value is very close to 0]. The first reaction order with respect to the alkyne suggests that the insertion of the latter into the Pt-H bond is the rate-determining step, in agreement with the recent results reported by Kuhn’s group [45]. These two kinetically monitored reactions at 0.5 h show the respective turnover frequencies (TOF) are 174 and 198 h^−1^. These data show that **2A** is recoverable, robust and catalytically effective.

**Scheme 3:**
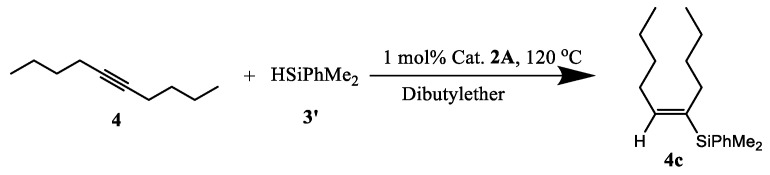
The Pt catalyst **2A**-catalyzed hydrosilylation of 5-decyne (**4**) with dimethylphenyl-silane (**3′**) under thermomorphic condition.

The Pt catalyst **2A**-catalyzed hydrosilylation of 5-decyne (**4**) and dimethylphenylsilane (**3′**) was also carried out in a shorter reaction time (ca. 40 min) to afford only E-form product of **4c** based on the GC-MS analysis, as shown in Scheme 3. The good recycling results under the thermomorphic condition were also confirmed by the nearly quantitative conversion of the hydrosilylation catalyzed by the reused Pt catalyst for up to eight cycles (Table 2) with very good turnover number (TON = ~100).

(b) Terminal alkynes

b.1. Two terminal alkyl acetylenes

**Scheme 4:**
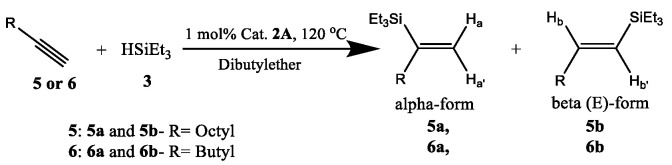
The Pt complex **2A**-catalyzed hydrosilylation of 1-decyne (**5**) or 1-hexyne (**6**) with triethylsilane (**3**) under thermomorphic condition.

The Pt complex **2A**-catalyzed hydrosilylation of triethylsilane (**3**) with terminal alkyne [i.e., 1-decyne (**5**) or 1-hexyne (**6**)] under thermomorphic mode is shown in Scheme 4. The hydrosilylation of 1-decyne (**5**) with triethylsilane (**3**) afforded two products, triethyl(dec-1-en-2-yl)silane (**5a**) and (*E*)-triethyl(dec-1-en-1-yl)silane (**5b**) with a GC-MS or NMR method to monitor the reaction and to determine the products under thermomorphic condition. This hydrosilylation also showed very good recycling results under thermomorphic condition. The structure of **5a** and **5b** were identified by the ^1^H-NMR spectrum of their mixture (see Figure S7). The signals of H_a_ and H_a’_ of **5a** are shown as two doublet peaks at 5.2 and 5.6 ppm with a coupling constant of 2.7 Hz, which is consistent to that of geminal alkenyl protons. For **5b**, the H_b’_ shows a doublet-triplet splitting peak and the H_b_ shows a doublet peak with a coupling constant of 18.9 Hz, which represents for the alkenyl protons at the *trans* position. The ratio of **5a** and **5b** determined from the integration of ^1^H-NMR spectrum equals to 1:2, which is almost similar to the ratio from GC-MS method (1:1.82) (see entry 5 in Table 3).

The other terminal alkyne, 1-hexyne (**6**) also underwent the hydrosilylation reaction with triethylsilane (**3**) in the presence of Pt catalyst **2A** successfully (Scheme 4). The reaction was completed within 30 min to produce two isomers α-form **6a** and β-(*E*)-form **6b** whose ^1^H-NMR spectrum shows similar characteristic peaks (see Figure S8) to that of α-form **5a** and β-(*E*)-form **5b**. The signals for H_a_ and H_a’_ of **6a** are 5.3 ppm (dt, *J* = 3.3, 1.2 Hz) and 5.6 ppm (dt, *J* = 3.3, 1.5 Hz); and the signals for H_b_ and H_b′_ of **6b** are 5.5 ppm (dt, *J* = 18.6, 1.5 Hz) and 6.0 ppm (dt, *J* = 18.6, 6.3 Hz), respectively. As shown in entry 1 of Table S1, the ratio of **6a** and **6b** is approximately 1:4.7 estimated by a GC-MS method.

b.2. One functionalized terminal alkyl acetylene (propargyl alcohol)

**Scheme 5:**
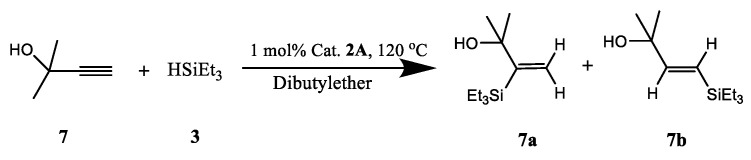
The Pt-catalyzed hydrosilylation reaction of 2-methylbut-3-yn-2-ol (**7**) and triethylsilane (**3**) under thermomorphic condition.

In Scheme 5, the hydrosilylation of 2-methylbut-3-yn-2-ol (**7**), a terminal alkyne with a bulky substituent (hydroxyisopropyl) with triethylsilane (**3**) for 1 h afforded two isomers, α-form **7a** and β-(*E*)-form **7b** in 99.4% yield with an estimated ratio of 1:2.1 from GC data, and/or 1:2.3 from ^1^H-NMR spectra. The good recycling results for this reaction were shown in Table 4. When comparing with the previously reported long fluorous chained platinum complex (α:β = 1:2) [19], a slightly better selectivity was observed in this work by using Pt catalyst **2A** (1:2.3). Although the heterogeneous catalyst PtO/PtO_2_-Fe_3_O_4_ in literature [46] showed a better selectivity for **7a** and **7b** (1:3.5), it is worth mentioning that the Pt complex **2A** in this work still has good selectivity among a homogeneous type of catalysts.

(c) Three terminal aryl acetylenes

**Scheme 6:**
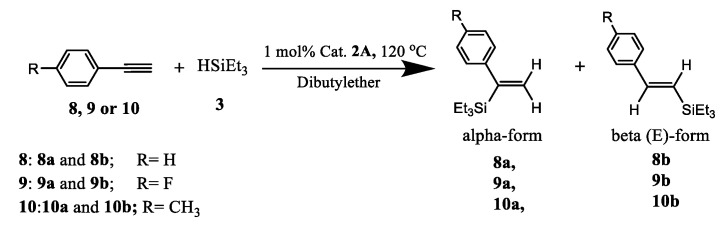
The Pt complex **2A**-catalyzed hydrosilylation of phenyl acetylenes (**8**~**10**) with triethyl-silane (**3**).

In Scheme 6, the Pt catalyst **2A**-catalyzed hydrosilylation of phenylacetylene (**8**) with triethylsilane (**3**) for 1~3 h under thermomorphic condition afforded two isomers, α-form **8a** and β-(*E*)-form **8b** with an estimated ratio of 2:1 according to GC-MS analysis. The characteristic ^1^H-NMR signals (see Figure S9**)** for the geminal alkenyl protons of α-form **8a** appear at 5.6 ppm (d, *J* = 3 Hz) and 5.9 ppm (d, *J* = 3 Hz), and the signals for the trans position protons of (*E*)-form **8b** show at 6.4 ppm (*J* = 18.9 Hz) and 6.9 ppm (*J* = 19.5 Hz)**.** Most of the ratios of **8a** and **8b** determined by the integration of ^1^H-NMR spectrum are close to 2:1.

Which is consistent to the ratio from GC-MS data in Table 5. Interestingly, the major product for the hydrosilylation of the terminal aryl acetylene (**8**) is thermodynamically unfavored α-form **8a**, whereas the major products for the alkyl acetylenes (**5**, **6** and **7**) are thermodynamically favored β-(*E*)-form **5b**, **6b** and **7b**. The selectivity of ratio of **8a**:**8b** decreased after 6 runs is because some of the catalysts start to degrade during the course of repeated reactions.

When we compare the efficiency of our catalyst **2A** with a heterogeneous molecular complex immobilized on the surface of graphene, catalyst **2A** showed better catalytic efficiency at the elevated temperature. Mata [35] and his colleagues used 1 mol% catalytic loading of their catalyst in the similar reactions to obtain 40% yield after 3 h where TON and TOF are 40 and 13, respectively. They have further optimized the reaction and used 0.5 mol% loading to obtain 94% yield after 20 h. Where TON and TOF are 188 and 9.4, respectively. However, we have obtained on average 100% yield in 2 h with TON and TOF values of 100 and 50, respectively.

According to the literatures, which isomer being the major product for the hydrosilylation of phenylacetylene depends on the different catalysts. The thermodynamically stable β-(*E*)-isomer was the major product when the hydrosilylation was catalyzed with a homogeneous Karstedt’s catalyst at 60 °C for 1 h, and the product ratio was β-(*E*):β-(*Z*): α isomers = 81:1:18 [30]. However, the α isomer was the major product when it was catalyzed by a heterogeneous catalyst Pt/CeO_2_ at 70 °C for 18 h [44] with the product ratio of β-(*E*):α = 26:74 and the conversion 39% for THF and 50% for water as the solvents, respectively. Comparatively, using the Pt catalyst **2A** in this work which was homogeneous under thermomorphic mode, but provided thermodynamically unfavored α-form as the major product with better conversions (mostly more than 99%) and shorter reaction time (1~3 h).

**Figure 1:**
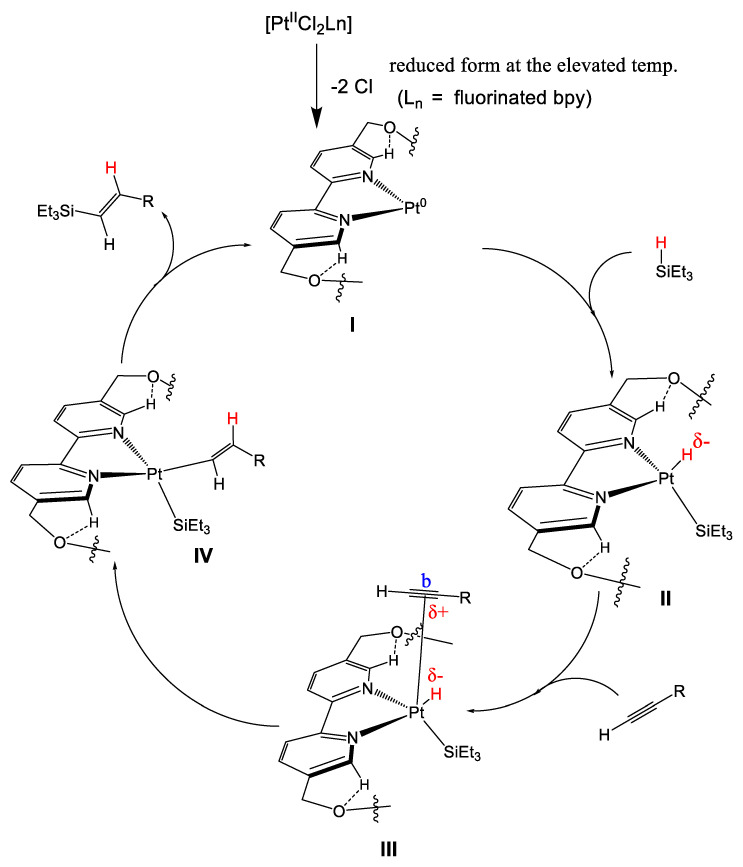
The proposed mechanism for **2A**-catalyzed hydrosilylation of terminal (alkyl) alkyne with triethylsilane. (Note: H is labeled in red color).

Lastly, to further understand this unusual product selectivity, two more substituted phenyl acetylenes with electron-withdrawing fluoro group and electron-donating methyl group were brought to this **2A** catalyzed hydrosilylation under thermomorphic mode. The hydrosilylation of 4-fluorophenylacetylene (**9**) at 120 °C for 4~5 h afforded α-form isomer as the major product in 99% high yield with the product ratio of **9a**:**9b** = 2.54:1 (estimated by GC) (Table S2). Similarly, the hydrosilylation of 4-methylphenylacetylene (**10**) at 120 °C for 2~5 h also produced α-form isomer as the major product in 99% high yield with the product ratio of approximately **10a**:**10b** = 2.13:1 estimated by GC (entry 4, Table S3). The results of hydrosilylation reactions of either 4-fluorophenylacetylene (**9**) or 4-methylphenylacetylene (**10**) with triethylsilane (**3**) under thermomorphic conditions are shown Tables S2 and S3 in Supplementary Materials. No matter what kind of three substituents connected on the phenyl ring at para position, the thermodynamically less favored α-form isomer would be the major product. Thus, Figure 1 and the related discussion below also explain the observed moderate regioselectivity of the **2A**-catalyzed hydrosilylations of aryl and aliphatic acetylenes.

To understand the better product ratio from the Pt complex **2A**-catalyzed hydrosilylation (short-fluorous-chains), a plausible mechanism containing the geometry of Pt complex was proposed. The optimized structure of **2A** in the gaseous phase has been obtained by DFT theoretical calculation, showing the two fluorous side-chains whose -CH_2_OCH_2_-R_f_ moieties can form the two five-membered rings at the both sides of 5,5′-substituted bipyridine to extend the metal planarity (in Figure 1 and Figure S10 of Supplementary Materials). Similar type of extended the Pt metal planarity (with U-shaped like arrangement) has been found on the experimental data of 55–8FH-PtI2 (see Figure S11) in our previous research [47]. This optimized 2A arrangement also shows the intramolecular five-membered ring and other weak interactions (see Figure S10); and also be observed to be similar to that of the complexes 55–4FH-MX2, where X = Br or I; M = Pt, with the loop structure reported by our group [48] (see Figure S12).

As shown is Figure 1 which is similar to Chalk-Harrod mechanism, the proposed mechanism for **2A**-catalyzed hydrosilylation of terminal aliphatic alkyne with triethylsilane is described in a 3D-view instead of 2D-view of a well-known Chalk-Harrod mechanism. Firstly, in species I the **2A** catalyst is believed to appear in the reduced form, Pt(0) [49] at the elevated temperature. Besides the planar Pt(bpy) core, the Pt complexes which contain two intramolecular five-membered ring in the species I, II, III and IV can extend its planarity with slight U-shaped structure. The Et_3_SiH is oxidatively added to [Pt], followed by coordination of the alkyne and insertion to Pt-H bond. Thus, the electronic effect due to the polarization of an alkyne in hydrosilylation would direct the hydride addition to the more electropositive C-atom (in II). After the oxidative addition of silane, the alkyne is shown by approaching from the top of the Pt(bpy)-based molecular plane. When the alkyne (the 2nd reactant) has been attached to the Pt, this step of migration-insertion shows that the R group of the alkyne (III at bottom) has been forced to keep away from the silyl-group side of the metal complex (in IV) which then leads to generate β-(*E*) form as the major product. This addition shows the anti-Markovnikov’s selectivity. This proposed mechanism reasonably explains the better product ratio of β-(*E*) to α in 1-decyne (**5**), 1-hexyne (**6**) and propargyl alcohol (**7**) in the hydrosilylations catalyzed by Pt catalyst **2A** in this study. However, if the R group is the aromatic moiety, then the bond axis of the linear aryl acetylene molecule (H-C≡C-R) rotates more than 90^o^ with respect to Pt-b axis (which is the molecular plane normal) (see Figure 1). For example, in an extreme case, if the tilting angle is equal to 180°, then it is like the linear molecule is exactly flipped over (R-C≡C-H). Such a phenomenon occurs possibly due to steric reason and other weak interactions (e.g., pi-pi interaction), when linear aromatic alkyne in species III is attached to the Pt center. Thus, **2A**-catalyzed hydrosilylation of terminal aryl acetylenes with triethylsilane gives rise to the α isomer as the major product.

### 2.3. Detection of Metal Recovery by ICP-MS

After discussing the hydrosilylation reaction of alkyne substrates, we randomly used ICP-MS to detect the leakage of metal catalysts, as shown in Table 6. The quantity of residual Pt metal present in the solution was analyzed, taking random samples among the product solutions resulting from the thermomorphic hydrosilylation. Only very low Pt leaching was observed in solution from the complex **2A**- and **2B**-catalyzed hydrosilylations under thermomorphic conditions. The residual platinum content in the product is only minimal since the recovery rate is greater than 99 %. Looking at the leaching test results, whether it is for **2A** or **2B**, the measured platinum content is in the ppb level even after the 8 times of repeated recycling. It can be concluded that the metal catalyst used in this experiment not only effectively catalyzes hydrosilylation, but also easily achieved with almost no Pt leaching during the recycling processes.

In order to evaluate the effect of a series of fluorous chain on the Pt catalyst recovery, the fluorinated moieties of R_f_ = CF_3_, C_4_F_8_Cl (C_4_F_8_H), C_8_F_16_H and C_11_F_23_ in the fluorous chain have been investigated for the systematic purpose. In other words, for the fluorous chains with R_f_ = CF_3_ and C_8_F_16_H moieties which are from respective [PtCl_2_(5,5′-bis(CH_2_OCH_2_CF_3_)-2,2′-bpy)] (**2C**) and [PtCl_2_(5,5′-bis(CH_2_OCH_2_C_8_F_16_H)-2,2′-bpy)] (**2D**) complexes have also studied to give a trend for the series (see Section S7 in Supplementary Materials). When R_f_ = CF_3_ group, it has been found that **55-3F-PtCl_2_** (**2C**) is totally soluble in DMF and moderately soluble in Bu_2_O. The images of these solubility properties are shown in Section S7 of Supplementary Materials (see Figure S13a,b). Thus, complex **2C** with one carbon fluoro-chain which is so short that it cannot give the useful heterogeneous phase separation property at room temperature for the recycling studies here. When R_f_ = C_8_F_16_H group which is sparingly soluble in Bu_2_O ether (solubility image is shown in Figure S14 in Supplementary Materials).

Thus, **55-16FH-PtCl_2_**-catalyzed hydrosilylations of 5-decyne with HSiEt_3_ at 120 °C under thermomorphic mode have also been carried out. These results shown in Table S4 in Supplementary Materials indicate that these **55–16FH-PtCl_2_** (**2D**)-catalyzed hydrosilylations can take slightly longer than 3 h to complete. Thus, by comparing the reactivities from a series of Pt metal complexes which are **55-8FCl-PtCl_2_** (**2A**), **55-16FH-PtCl_2_** (see Section S7.2, Table S4 in Supplementary Materials) and **55-23F-PtCl_2_** [19], the rate due to the steric reasons follows the trend: the shorter the chain of fluorous catalyst, the faster the hydrosilylation.

## 3. Experimental Section

### 3.1. General Procedures

HP 6890 GC containing a 30 m 0.250 mm HP-1 capillary column with a 0.25 mm stationary phase film thickness was used to censor the reaction. The same GC instrument with a 5973 series mass selective detector was used to Acquire GC/MS data. The flow rate was 1 mL/min and splitless. Samples analyzed by fast atom bombardment (FAB) mass spectroscopy were done by the staff of the National Central University (Taoyuan, Taiwan) mass spectrometry laboratory. The amounts of residual Pt samples were analyzed by ICP-MS. Infra-red spectra were obtained on a Perkin Elmer RX I FT-IR Spectrometer. NMR spectra were recorded on Bruker AM 500 and Joel AM 200 using 5 mm sample tubes. CD_3_OD, CD_2_Cl_2_, CDCl_3_, deuterated DMF and deuterated DMSO were the references for both ^1^H- and ^13^C-NMR spectra; and Freon^®^ 11 (CFCl_3_) was the reference for ^19^F NMR spectra.

### 3.2. General Procedures in Catalytic Hydrosilylation Reaction and Recovery

In a typical run, an alkyne (2 mmol) and silane (2 mmol) were charged into 10 mL reaction tube containing a magnetic stirrer bar, then the Pt catalyst (1 mol%) and followed by dibutyl ether, DBE, solvent (2 mL). Then the reaction mixture was set to react at 120 °C for the given period of time before GC/MS analysis was done to confirm the completion of the reaction. Once the catalytic recovery was done, the pure product was isolated by using CH_2_Cl_2_/water extraction and the CH_2_Cl_2_ layer was pumped under reduced pressure to obtain the pure product. The product was finally analyzed by using ^1^H-NMR spectroscopy.

The Pt-catalyzed hydrosilylation reactions of all the alkyne substrates with **3** were carried out under the thermomorphic condition that effectively confirmed the practicability of reutilizing the catalyst in Bu_2_O solvent. Every round the catalytic reaction was performed at 120 °C under N_2_ gas. At the end of each run, the product mixtures were put into freezer (0 °C) for about 5 min, and then centrifuged to separate the catalyst and reaction mixture (see Scheme S1 in Supplementary Materials). After catalyst was recovered by decantation and washed three times by the same solvent, it was again supplied with the same amounts of Bu_2_O solvent, the alkyne substrate, and HSiEt_3_ to continue to the next round. The products were tracked with GC-MS and ^1^H-NMR, using NMP as internal standard until the end of the reaction was confirmed.

### 3.3. Starting Materials

Chemicals, reagents, and solvents employed were commercially available and used as received. H(CF_2_)_4_CH_2_OH and Cl(CF_2_)_4_CH_2_OH were purchased from either Aldrich or SynQuest.

### 3.4. Preparation of Platinum Complexes

The reaction of K_2_PtCl_4_ with fluorinated bipyridine derivatives, bis-5,5′-(*n*-C_4_F_8_ClCH_2_OCH_2_)-2,2′-bpy (**1A**) and bis-5,5′-(*n*-C_4_F_8_HCH_2_ OCH_2_)-2,2′-bpy (**1B**), resulted in the synthesis of PtCl_2_[5,5′-bis-(*n*-C_4_F_8_ClCH_2_OCH_2_)-2,2′-bpy] (**2A**) and PtCl_2_[5,5′-bis-(*n*-C_4_F_8_HCH_2_OCH_2_)-2,2′-bpy] (**2B**), as red solids. **1A** or **1B** (10.0 mmol; its synthesis see Supplementary Materials), K_2_PtCl_4_ (11.0 mmol) and DMSO (2 mL) were charged into 50 mL round-bottomed flask. The reaction was stirred under nitrogen at room temperature for 4 h, and the complexes **2A** and **2B** would then precipitate from the reaction mixture [50,51,52,53]. After collection of the precipitates, water and Et_2_O were used to wash the red solids for several times, and the yields were 87% and 85%, for **2A** and **2B,** respectively.

### 3.5. Analytical Data for Two Ligands and Their Platinum Metal Complexes (***1A***, ***1B***, ***2A***, ***2B***)

***55-8FCl-bpy* (1A).** Yield: 90%. m.p.: 75 °C. FT-IR (cm^−1^): *v* (bpy, m) 1600, 1557, 1466; *v* (CF_2_ stretch, s) 1182, 1112. ^1^H-NMR (300 MHz, DMSO-d_6_, rt): δ (ppm) 8.65 (d, *J* =1.5 Hz, 2H, H6), 8.39 (d, *J* = 8.4 Hz, 2H, H3), 7.90 (dd, *J* = 8.1 Hz, *J* = 2.1 Hz, 2H, H4), 4.78 (s, 4H, bpy-CH_2_), 4.28 (t, *J* = 7.35 Hz, 4H, -CH_2_CF_2_-); ^13^C-NMR (125 MHz, DMSO-d_6_, 350 K): δ (ppm) 192.3, 186.2, 174.3, 170.5, 157.8 (10C, bpy), 161.6–144.2 (-C_4_F_8_Cl), 108.4 (bpy-CH_2_O-), 103.9 (-CH_2_CF_2_-); ^19^F-NMR (470 MHz, DMSO-d_6_, 350 K): δ (ppm) −68.4 (t, *J* = 14.1 Hz, 4F, -CF_2_Cl), −118.8 (t, 4F, -CH_2_CF_2_), −120.2 (m, 4F, -CF_2_CF_2_Cl), −122.1 (s, 4F, -CH_2_CF_2_CF_2_-). 713 (M^+^), 463 (M^+^-CH_2_C_4_F_8_H), 447 (M^+^-OCH_2_C_4_F_8_H), 182 (M^+^-(OCH_2_C_4_F_8_H)_2_), 91 (C_6_H_5_N).

***55-8FH-bpy* (1B).** Yield: 80%. m.p.: 68 °C. FT-IR (cm^−1^): *v* (bpy, m) 1598, 1558, 1469; *v* (CF_2_ stretch, s) 1159, 1126. ^1^H-NMR (300 MHz, CDCl_3_, rt): δ (ppm) 8.62 (s, 2H, H6), 8.41 (d, *J* = 8.2 Hz, 2H, H3), 7.80 (d, *J* = 8.2 Hz, 2H, H4), 6.04 (tt, *J* = 51.9 Hz, *J* = 5.5 Hz, 2H, CF_2_H), 4.70 (s, 4H, bpy-CH_2_), 3.99 (t, *J* = 14.68 Hz, 4H, -CH_2_CF_2_-); ^13^C-NMR (125 MHz, CDCl_3_, 350 K): δ (ppm) 121.0, 132.1, 136.5, 148.6, 155.9 (10C, bpy), 108.0–118.5 (-C_4_F_8_Cl), 71.9 (bpy-CH_2_O-), 67.0 (-CH_2_CF_2_-); ^19^F-NMR (470 MHz, CDCl_3_, 350 K): δ (ppm) −137.2 (t, *J* = 13.7 Hz, 4F, -CF_2_H), −119.5 (t, 4F, -CH_2_CF_2_), −125.4 (m, 4F, -CF_2_CF_2_Cl), −130.2 (s, 4F, -CH_2_CF_2_CF_2_-). GC/MS: 644 (M^+^), 429 (M^+^-CH_2_C_4_F_8_H), 413 (M^+^-OCH_2_C_4_F_8_H), 182 (M^+^-(OCH_2_C_4_F_8_H)_2_), 91 (C_6_H_5_N).

***55-8FCl-PtCl_2_* (2A).** Yield: 87%. Decomposed temperature = 315.5 °C. FT-IR (cm^-1^): *v* (bpy, m) 1617, 1477; *v* (CF_2_ stretch, s) 1180, 1132, 1103. ^1^H-NMR (500 MHz, DMSO-d_6_, rt): δ (ppm) 9.47 (d, *J* =1.8 Hz, 2H, H6), 8.56 (d, *J* = 8.1 Hz, 2H, H3), 8.32 (dd, *J* = 8.7 Hz, *J* = 1.8 Hz, 2H, H4), 4.92 (s, 4H, bpy-CH_2_), 4.36 (t, *J* = 14.1 Hz, 4H, -CH_2_CF_2_-); ^13^C-NMR (125 MHz, DMSO-d_6_, 350 K): δ (ppm) 155.8, 146.9, 138.7, 137.2, 123.6 (10C, bpy), 148.3–120.1 (-C_4_F_8_Cl), 70.1 (bpy-CH_2_O-), 66.8 (-CH_2_CF_2_-); ^19^F-NMR (470 MHz, DMSO-d_6_, 350 K): δ (ppm) −67.9 (t, *J* = 14.1 Hz, 4F, -CF_2_Cl), -118.5 (t, 4F, -CH_2_CF_2_), −119.7 (m, 4F, -CF_2_CF_2_Cl), -121.6 (s, 4F, -CH_2_CF_2_CF_2_-). HR-FAB: (M^+^, *m*/*z*): C_22_H_14_^35^Cl_4_F_16_N_2_O_2_Pt calculated: 976.9202, found: 976.9207; C_22_H_14_^35^Cl_3_^37^ClF_16_N_2_O_2_Pt calculated: 978.9185, found: 978.9183; C_22_H_14_^35^Cl_2_^37^Cl_2_F_16_N_2_O_2_Pt calculated: 980.9185, found: 980.9171.

***55–8FH-PtCl_2_* (2B).** Yield: 85 %. m.p.: 290 ℃. FT-IR (cm^−1^): *v* (bpy, m) 1672, 1477; *v* (CF_2_ stretch, s) 1394, 1164, 1108. ^1^H-NMR (500 MHz, DMSO-d6, rt): δ (ppm) 9.49 (s, H6), 8.57 (2H, d, *J* = 8.2 Hz, 2H, H3), 8.33 (d, *J* = 8.2 Hz, 2H, H4), 7.05 (tt, *J* = 50.3 Hz, *J* = 5.6 Hz, 2H, HCF_2_-), 4.94 (4H, s, bpy-CH_2_), 4.33 (4H, t, *J* = 14.8 Hz, -CH_2_CF_2_-); ^13^C-NMR (125 MHz, DMSO-d6, rt): δ (ppm) 156.0, 146.9, 139.0, 137.4, 123.9 (10C, s, bpy), 120.0–105.0 (8C, -C_4_F_8_H), 70.1 (2C, s, bpy-CH_2_), 66.6 (2C, t, *J* = 24.5 Hz, -CH_2_CF_2_-); 19F-NMR (470 MHz, DMSO-d6, rt): δ(ppm) −119.7 (m, 4F, -CH_2_CF_2_-), −125.1 (s, 4F, -CH_2_CF_2_CF_2_-), −130.1 (m, 4F, -CF_2_CF_2_H), −138.8 (d, 4F, *J* = 49.6 Hz, -CF_2_H). HR-FAB: (M^+^, *m*/*z*) C_22_H_16_^35^Cl_2_F_16_N_2_O_2_Pt calculated *m*/*z* 908.9981, found 908.9985; C_22_H_16_^35^Cl^37^ClF_16_N_2_O_2_Pt calculated m/z 910.9952, found 910.9946; C_22_H_16_^37^Cl_2_F_16_N_2_O_2_Pt calculated *m*/*z* 912.9922, found 912.9921.

## 4. Conclusions

Concerns for the environment and scarcity of resources is becoming a challenge which motivates chemists to look for more economical and more ecofriendly process. Therefore, developing a recoverable catalyst with a better activity, better selectivity, and easier separation, with a better yield is everyone’s goal. Reported here, this work showed effective catalysis by using shortest fluoro-chain complexes containing the (%)F-content being less than 35 wt.%. The catalyst was very effectively recycled up to eight times for all the experiments done, but almost without affecting its activity and selectivity. In these reactions the thermomorphic mode was effectively used to recover these short fluorous ponytailed catalysts from the reaction mixture up to eight cycles by using heterogeneous phase separation at 0 °C. The product yield was also higher in every reaction run; sometimes it reached to even 100%. The metal leaching studies via ICP-MS method showed that the metal in the complex is effectively recovered throughout the catalytic cycles with very little loss, which is almost negligible. Additionally, the kinetic studies have been carried out for **2A**-catalyzed hydrosilylations of 5-decyne (**4**) with triethylsilane (**3**) in Bu_2_O at 120 and 130 °C. The results showed that the 1st order kinetics where the rate is directly proportional to the concentration of 5-decyne. Thus, we suggest that the insertion of alkyne into the Pt-H bond is the rate-determining step which agrees well with recent results reported by Kuhn et al. [45]. Furthermore, the regioselectivity followed the Chalk-Harrod mechanism as it undergoes the *syn* kind of reaction to give α and β-(*E*) products, of which the β-(*E*) was the major form for most of the cases.

## Figures and Tables

**Table 1:** Hydrosilylation reactions of 5-decyne (**4**) and triethylsilane (**3**) in the presence of **2A** and **2B** catalysts.

	55-8FCl-PtCl_2_ (2A)		55-8FH-PtCl_2_ (2B)	
Run	Time (h)	Yield (%) ^a^	Time (h)	Yield (%) ^a^
1	2	100	2	100
2	2	99 (89)	2	100
3	2	100 (94)	2	94
4	2	100	2	100 (91)
5	2	100	2	100 (94)
6	2	100	2	90
7	2	100	2	100
8	2	100	2	96

Reaction conditions: Temp = 120 °C; cat **2A** or **2B** 1 mol%, **4** (276 mg, 2 mmol), **3** (232 mg, 2 mmol), solvent: 2 mL. a: Based on GC yield; isolated yields are given in parentheses.

**Table 2:** Hydrosilylation reactions of 5-decyne (**4**) and dimethyl-phenylsilane (**3′**) under thermomorphic condition.

Cycle	Time (h)	Yield (%) ^a^
1	1	100
2	1	98
3	1	100 (97)
4	1	100
5	1	100
6	1	100
7	1	100 (97)
8	1	100

Reaction conditions: T = 120 °C, cat. 2A: 1 mol%, 4 (276 mg, 2 mmol), 3′ (272 mg, 2 mmol), solvent: 2 mL. a: Based on GC yield; isolated yields are given in parentheses.

**Table 3:** Hydrosilylation reaction of 1-decyne (**5**) and triethylsilane (**3**) under thermomorphic condition.

Cycle	Time (h)	5a:5b	Yield (%) ^a^
1	1	1:1.58	100
2	1	1:1.67	100 (96)
3	1	1:1.48	100 (92)
4	1	1:1.77	100
5	1	1:1.82	100 (92)
6	1	1:1.74	100
7	1	1:1.40	100 (99)
8	1	1:1.58	100 (91)

Reaction conditions: T = 120 °C, cat. **2A** 1 mol%, 5 (276 mg, 2 mmol), 3 (232 mg, 2 mmol), solvent: 2 mL. a: Based on GC yield; isolated yields are given in parentheses. The other terminal alkyne (1-hexyne (6)) recycling data are shown in Table S1 in Supplementary Materials.

**Table 4:** Hydrosilylation reaction of 2-methylbut-3-yn-2-ol (**7**) and triethylsilane (**3**) under thermomorphic condition.

Cycle	Time (h)	7a:7b	Yield (%) ^a^
1	1	1:2.1	100 (96)
2	1	1:2.1	99
3	1	1:2.0	99 (92)
4	1	1:2.2	99
5	1	1:2.1	98
6	1	1:1.9	100
7	1	1:2.0	100
8	1	1:2.0	99

Reaction conditions: T = 120 °C, cat. 2A 1 mol%, 7 (168 mg, 2 mmol), 3 (232 mg, 2 mmol), solvent: 2 mL. a: Based on GC yield; isolated yields are given in parentheses.

**Table 5:** Hydrosilylation reaction of phenylacetylene (**8**) and triethylsilane (**3**) under thermomorphic condition.

Cycle	Time (h)	8a:8b	Yield (%) ^a^
1	2	2.02:1	99 (92)
2	2	2.25:1	99 (96)
3	2	2.26:1	99
4	2	2.25:1	100 (98)
5	2	2.21:1	100
6	2	2.20:1	100
7	2	1.86:1	100
8	2	1.80:1	100

Reaction conditions: T = 120 °C, cat.2A 1 mol%, 8 (204 mg, 2 mmol), 3 (232 mg, 2 mmol), solvent: 2 mL. a: Based on GC yield; isolated yields are given in parentheses.

**Table 6:** Detection of platinum metal leakage by ICP-MS.

Table (& Cycle)	Catalyst	Ppb ^a^	Leaching Level (10^–4^) ^b^	Recovery (%)
1–2	**2A**	517.5	6.63	99.93
1–3	**2A**	444.7	5.70	99.94
1–7	**2A**	137.1	1.76	99.98
1–8	**2A**	41.6	0.53	99.99
1–2	**2b**	21.7	0.28	99.99
1–3	**2B**	31.3	0.40	99.99
1–7	**2B**	56.6	0.72	99.99
1–8	**2B**	43.6	0.56	99.99
2–4	**2A**	29.9	0.38	99.99
3–2	**2A**	107.5	1.38	99.98
3–4	**2A**	79.4	1.02	99.98
3–6	**2A**	60.7	0.78	99.99
S1–4 ^c^	**2A**	98.0	1.26	99.98
S1–7 ^c^	**2A**	1753.0	22.50	99.77
S1–8 ^c^	**2A**	2347.0	30.10	99.69
4–4	**2A**	276.5	3.54	99.96
5–4	**2A**	805.7	10.30	99.89

a: (in ppb) detected from ICP-MS analysis for every 5 mL of sample solution which is diluted from 2 mL reaction mixture. b: The Pt leaching level is calculated as follows: taking entry 1–2 (Table 1- Cycle 2) for example: (517.5 ppb × 5 mL)/(0.02 mmol Pt) = 517.5 × (mg Pt/103 L) × 5 × (10–3 L)/(2 × 10–2 × 195 mg Pt) = 6.63 × 10^−4^. c: These three entries are shown in Table S1 in Supplementary Materials.

## Data Availability

The data presented in this study are available in the article and supplementary materials.

## Supplementary Materials

The following are available online at, Figures S1–S4: thermomorphic property data, Figures S5 and S6: kinetic study, Figures S7–S9 ^1^H-NMR spectra of hydrosilylation products, Figures S10–S12: optimized structure from theoretical calculations, Tables S1–S4: recovery and recycling study. Scheme S1: The recycling procedure of the complex **2A** during the recoverable **2A**-catalyzed hydrosilylation.

## Sample Availability

Some of fluorous platinum metal complexes are available from the authors.
