# Supplementary material for: Short-Chained Platinum Complex Catalyzed Hydrosilylation under Thermomorphic Conditions: Heterogeneous Phase Separation at Ice Temperature
Source: Molecules. 2021 Jan 13;26(2):378. doi: 10.3390/molecules26020378 (PMC7828357; doi:10.3390/molecules26020378)
Supplement: Supplementary file 1 [file molecules-26-00378-s001.pdf]

Short-Chained Platinum Complex Catalyzed Hydrosilylation under Thermomorphic Conditions: Heterogeneous Phase Separation at Ice Temperature

Chiao-Fan Chiu [1,2,†], Jinn-Hsuan Ho [3,†], Eskedar Tessema [4], Yijing Lu [4], Chia-Rui Shen [5,6], Chang-Wei Lin [7,*] and Norman Lu [4,8,*]

1 Department of Pediatrics, Linkou Medical Center, Chang Gung Memorial Hospital, Taoyuan 333, Taiwan; chioufan2008@gmail.com
2 Graduate Institute of Clinical Medical Sciences, College of Medicine, Chang Gung University, Taoyuan 333, Taiwan
3 Department of Chemical Engineering, National Taiwan University of Technology, Taipei 106, Taiwan; jhho@mail.ntust.edu.tw
4 Institute of Organic and Polymeric Materials, National Taipei University of Technology, Taipei 106, Taiwan; eskedartessema18@gmail.com (E.T.); yijinglu@hotmail.com.tw (Y.L.)
5 Department of Medical Biotechnology and Laboratory Sciences, College of Medicine, Chang Gung University, Taoyuan 333, Taiwan; crshen@mail.cgu.edu.tw
6 Department of Ophthalmology, Linkou Medical Center, Chang Gung Memorial Hospital, Taoyuan 333, Taiwan
7 Department of Internal Medicine, Division of Pulmonary and Critical Care Medicine, Chang Gung Memorial Hospital & Chang Gung University, Taoyuan 333, Taiwan
8 Development Center for Smart Textile, National Taipei University of Technology, Taipei 106, Taiwan
* Correspondence: naturewei@cgmh.org.tw (C.-W.L.); normanlu@mail.ntut.edu.tw (N.L.)
† These authors contributed equally to this work.

Supplementary Materials (SM)

1. **General Procedures** S2
2. **Plot of Solubility vs. Temperature** S4
3. **Kinetic Studies** S7
   - 3.1. **Kinetic Study at 120 °C**
   - 3.2. **Kinetic Study at 130 °C**
4. **NMR Spectra of Hydrosilylation Reaction Products** S9
   - 4.1. **NMR Spectrum of 5a and 5b**
   - 4.2. **NMR Spectrum of 6a and 6b**
   - 4.3. **NMR Spectrum of 8a and 8b**
5. **Tables (S1–S3) from Recycling Study** S11
6. **Computational Method** S13
7. **Probes of a Series of Fluorinated Pt Complexes** S16
   - 7.1. **Solubilities 55-3F-$PtCl_2$ in Bu_2O and DMF**
   - 7.2. **Solubility and reactivities of 55-16FH-$PtCl_2$**

7.3. of 55-16FH-$PtCl_2$

8. Schematic Recycling Drawing S18

9. References S19

1. Experimental (General Procedues)

HP 6890 GC containing a 30 m 0.250 mm HP-1 capillary column with a 0.25 mm stationary phase film thickness was used to censor the reaction. The same GC instrument with a 5973 series mass selective detector was used to Acquire GC/MS data. The flow rate was 1 mL/min and splitless. Samples analyzed by fast atom bombardment (FAB) mass spectroscopy were done by the staff of the National Central University (Taiwan) mass spectrometry laboratory. The amounts of residual Pt samples were analyzed by ICP-MS. Infra-red spectra were obtained on a Perkin Elmer RX I FT-IR Spectrometer. NMR spectra were recorded on Bruker AM 500 and Joel AM 200 using 5 mm sample tubes. CD_3OD, CD_2Cl_2, $CDCl_3$, deuterated DMF and deuterated DMSO were the references for both ^1H and ^{13}C NMR spectra; and Freon® 11 ($CFCl_3$) was the reference for ^{19}F-NMR spectra.

Preparation of other bipyridyl ligands and their platinum complexes

Synthetic procedure of the ligands[1]

R_fCH_2OH (11.0 mmol) and CH_3ONa (30% in methanol) (10.0 mmol) were charged into a N_2 filled two neck round-bottomed flask, then stirred continuously at 65 °C for 4 hours before methanol was vacuum pumped to facilitate the reaction conversion to the fluorinated alkoxide product. The fine powdered fluorinated alkoxide (10.0 mmol) was then dissolved in dry THF (30 mL), and 5,5'-bis($BrCH_2$)-2,2'-bpy (4 mmol) was added. The mixture was allowed to reflux for 4 h, and the completeness of the reaction was checked by sampling the reaction mixtures and analyzing the aliquots with GC/MS. Finally, the pure product was isolated by using CH_2Cl_2/water extraction to find a white solid material.

The reaction of K_2PtCl_4 with fluorinated bipyridine derivatives, bis-5,5'-(*n*-$R_fCH_2OCH_2$)-2,2'-bpy resulted in the synthesis of $PtCl_2$[5,5'-bis-(*n*-$R_fCH_2OCH_2$)-2,2'-bpy] as red solids. The reaction was stirred under nitrogen for 4 hours, and the complexes (also abbreviated as **55-no. of F's-$PtCl_2$**) would then precipitate from the reaction mixture. After collection of the

precipitates, water and Et_2O were used to wash the red solids for several times, and the yields were about 85%.

[Note: $PtCl_2$[5,5'-bis-(n-$R_fCH_2OCH_2$)-2,2'-bpy] are also abbreviated as **55-no. of F's-PtCl₂**.]

Analytical data of bis-5,5'-(*n*-$CF_3CH_2OCH_2$)-2,2'-bpy

55-3F-bpy. Yield: 75%. ^{1}H-NMR (500 MHz, d-DMSO): δ (ppm) 8.66 (2H, s, H-6), 8.39 (2H, d, $^3J_{H-H}$ = 8.24 Hz, H-4), 7.92 (2H, d, $^3J_{H-H}$ = 8.24 Hz, H-3), 4.77 (4H, s, bpy–CH_2), 4.17 (4H, q, $^3J_{H-F}$ = 9.34 Hz, CF_3CH_2). MS: 380.2 $[M]^+$, 297.1 $[M-CH_2CF_3]^+$, 281.1 $[M-OCH_2CF_3]^+$, 198.1 $[M-O(CH_2CF_3)_2]^+$, 182.1 $[M-(OCH_2CF_3)_2]^+$, 91.1 $[M-O(CH_2CF_3)_2C_6H_5N]^+$.

Analytical data of bis-5,5'-(*n*-$C_8F_{16}HCH_2OCH_2$)-2,2'-bpy

55-16F-bpy. Yield: 80 %. ^{1}H-NMR (300 MHz, d-DMSO): δ (ppm) 8.63 (2H, s, H-6), 8.41 (2H, d, $^3J_{H-H}$ = 8.1 Hz, H-4), 7.81 (2H, d, $^3J_{H-H}$ = 8.1 Hz, H-3), 6.04 (2H, tt, $^3J_{H-F}$ = 51.9 Hz, $^2J_{H-F}$ = 5.1 Hz, -CF_2H), 4.75 (4H, s, bpy-CH_2), 4.00 (4H, t, $^3J_{H-F}$ = 13.8 Hz, CH_2-CF_2). MS: 614.1 $[M-OCH_2C_8F_{16}H]^+$, 198.1 $[M-O(CH_2\ C_8F_{16}H)_2]^+$, 182.1 $[M-(OCH_2C_8F_{16}H)_2]^+$, 91.1 $[M-O(CH_2CF_3)_2C_6H_5N]^+$.

Analytical data of 55-3F-PtCl₂

Yield: 83 %. ^{1}H-NMR (300 MHz, DMSO): δ (ppm) 114 δ = 9.48 (2H, s, H6), 8.56 (2H, d, $^3J_{HH}$ = 8.2 Hz, H3), 8.35 (2H, d, $^3J_{HH}$ = 8.2 Hz, H4), 4.92 (4H, s, bpy-CH_2), 4.25 (4H, q, $^3J_{HF}$ = 9.2 Hz, -CH_2CF_3).

Analytical data of 55-16FH-PtCl₂. Yield 85 %. ^{1}H-NMR (300 MHz, d-DMSO): δ9.49 (2H, s, H-6), 8.58 (2H, d, $^3J_{H-H}$ = 8.4 Hz, H-4), 8.35 (2H, d, $^3J_{H-H}$ = 8.4 Hz, H-3), 7.21 (2H, tt, $^3J_{H-F}$ = 45.6 Hz, $^2J_{H-F}$ = 4.8 Hz , -CF_2H), 4.93 (4H, s, bpy-CH_2), 3.93 (4H, t, $^3J_{H-F}$ = 8.1 Hz, CH_2-CF_2).

2. Plot of Solubility vs. Temperature

The fluorous Pt-complex-catalyzed hydrosilylation can be carried out in dibutylether (DBE) or polar aprotic solvents (e.g DMF). In DMF, for the temperature variable NMR temprature range, its thermomorphic property verse temp relationship can be well monitored in the range of 0 and 80 °C. Thus, the thermomophic property of metal catalyst 55-8FCl-$PtCl_2$ (**2A**) has been studied in deuterated DMF.

The internal standard HOCH2C4F8H (46.4 mg, 0.2 mmol) and the metal catalyst **55-8FCl-PtCl2** (**2A**) (15 mg, 0.015 mmol) were added to 0.2 mL of d-DMF, and then filled into a glass tube with an outer diameter of 4 mm. The glass was flame-torched and the upper end of the tube is sealed. The 1H-NMR spectrum was measured between −5 °C and 80 °C at about 15 °C intervals as the integral ratio between the signal of the triplet-of-triplet peak of the terminal hydrogen of the internal standard and the signal of H6 on the platinum metal catalyst.

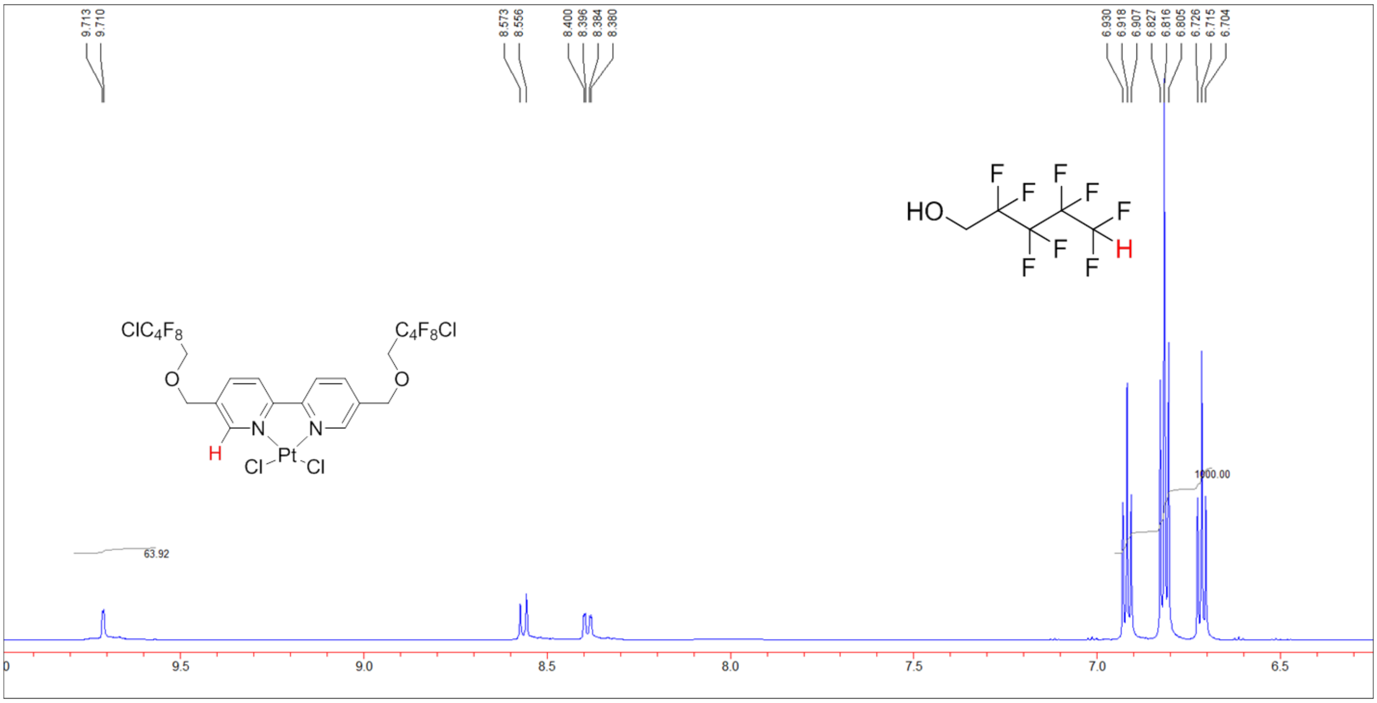

Figure S1. ^{1}H-NMR spectrum of **55-8FCl-$PtCl_2$** catalyst (**2A**) and $HOCH_2(CF_2)_4H$ at 80 °C.

As shown in Figure S1, the signal at 6.82 ppm is from the terminal hydrogen nine-peak of the internal standard $HOCH_2C_4F_8H$, while the signal at 9.71 ppm is H6 on a platinum metal catalyst. It is known that the concentration of the internal standard is 1000 mM, so the integrated value ratio of the terminal hydrogen of the internal standard to H6 can be used to determine the concentration of the metal catalyst, that is, its solubility, and measure the solubility of the

catalyst at different temperatures. It can be seen that the catalyst is still difficult to dissolve in organic solvents at lower temperature and soluble at high temperatures. The tendency of solubility of catalyst **2A** increasing with temperature has been observed by variable temperature NMR technique (shown in Figure S2).

For the Pt-catalyzed hydrosilylation reaction, it is usually carried out in ether. DBE has higher b.p., so it has also beenis chosen as a solvent for Pt-catalyzed hydrosylation reaction . In DBE, both complexes **2A** and **2B** are not soluble at room temperature. Thus, as reported here, complexes **2A** can be the efficienct and recoverable complex under thermomorphic mode for the Pt-catalyzed hydroslation reactions.

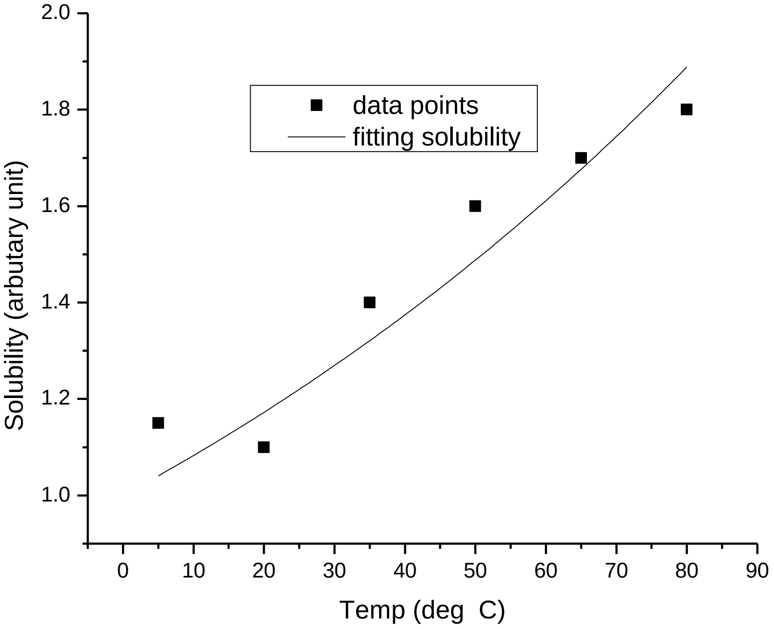

Figure S2. Solubility of **55-8FCl-PtCl$_2$** catalyst (**2A**) in DMF at different temperatures. The plot which is an exponential curve (curving up) shows the thermomorphic behavior of fluorinated complex, **2A**, in DMF.

Solubility of **55-8FCl-PtCl$_2$** catalyst (**2A)** shows the good thermomorphic behaviors mainly for polar aprotic solvents (e.g. DMF, DMSO and so on), but in DBE, its solubility dramatically increasing with temperature only appears when temperature needs to be higher than 80 °C. In DBE, the **55-8FCl-PtCl$_2$** catalyst (**2A) is insoluble at room temperature, but soluble at 120** °C (see Figures S3 and S4). [Note: The image of **2A** dissolved in DBE at 120 °C during catalysis is shown in Fig. S4 & Scheme S1.]

Because the temperature variable NMR spectrometer used can only heat up to 80 °C, we studied thermomorphic behavior of fluorinated complex (**2A)** mainly using deuterated DMF as a solvent[2,3](shown in Figure S2). In Figure S3, it can be seen that Pt complex **2A** is almost not solube in DBE when temp is below 40 °C. Due to the instrument limit of temperature variable NMR spectrometer used, only the slight thermomorphic behavior of **2A** in DBE has been recorded for the tempature up to 80 °C.

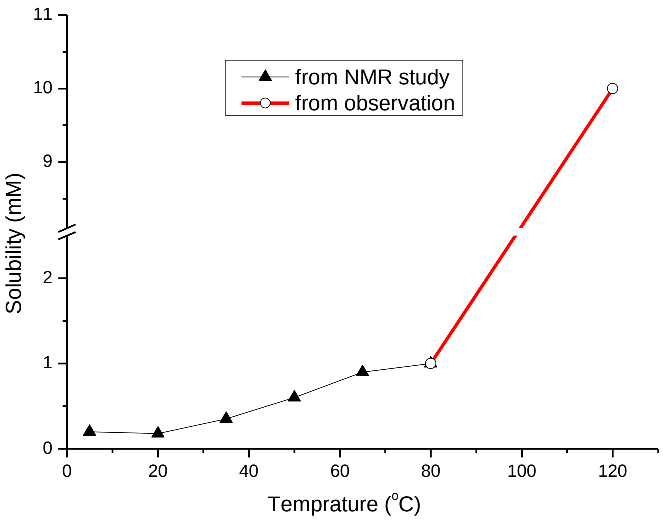

Figure S3. Solubility of **55-8FCl-PtCl$_2$** catalyst (**2A**) at different temperatures and its extrapolated solubility at 120 °C temperature in DBE solvent.

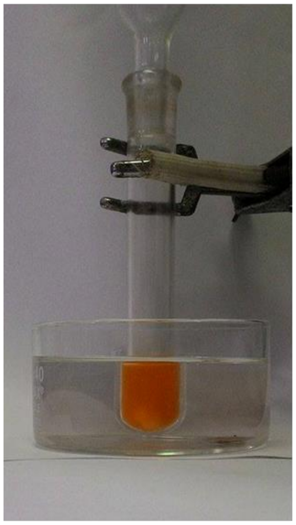

Figure S4. Hydrosilylation reaction mixture containing of **55-8FCl-PtCl$_2$** (**2A**) as a catalyst in Bu_2O at 120 °C temperature.

3. Kinetic Studies (Conversion as a Function of Time)

The 1 mol% of **2A** was used as the catalyst. The **2A-**catalyzed hydrosilylations of 5-decyne (**4**) with triethylsilane (**3**) in Bu_2O have also been studied kinetically and monitored at 120 and 130 °C for the set time intervals (5, 10, 15, 23, 30, 45 or 60 min)- by taking out small amounts of the reaction mixture for GC/MS analysis at the set intervals. The conversion was calculated from the GC/MS results using NMP as the internal standard.

The Pt catalyst **2A-**catalyzed hydrosilylations of 5-decyne (**4**) with triethylsilane (**3**) in Bu_2O have also been studied kinetically at 120 (Fig. S5 in Section S3.1) and 130 °C (Fig. S6 in Section S3.2). These two sets of data points are shown here. In both cases, the integrated rate law derived from the alkyne concentration vs time plots is ln[5-decyne] = ln[5-decyne]$_0$-kt, with k = 0.069 (R^2 = 0.935) & 0.099 (R^2 = 0.988) at 120 °C and 130 °C, respectively. [Note: ln[5-decyne]$_0$ = 0]; at 130 °C the derived ln[5-decyne]$_0$ value is very close to 0. The two respective rate constants derived now are 0.069 and 0.099. Thus, these two kinetically monitored reactions at 0.5 hr show the respective turnover frequencies (TOF) are 174 and 198 h^{-1}. These data show that **2A** is recoverable, robust and catalytically effective.

3.1. Kinetic study at 120 °C {[5-decyne]o = 1 M.}

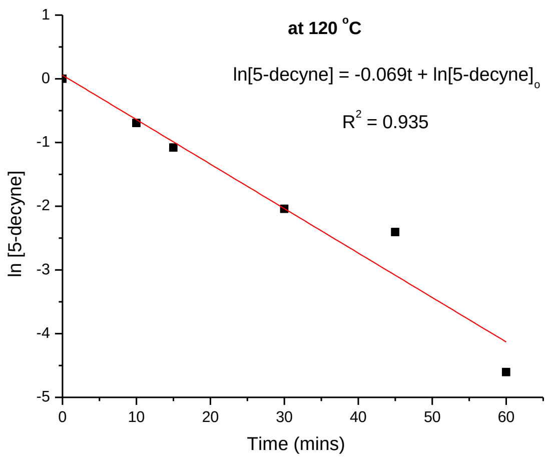

Time (min)	ln ([5-decyne])
0	0
10	−0.69
15	−1.08
30	−2.04
45	−2.41
60	−4.61

Figure S5. The linear plot of Pt catalyst **2A-**catalyzed hydrosilylations of 5-decyne (**4**) at 120 °C.

3.2. Kinetic Study at 130 °C

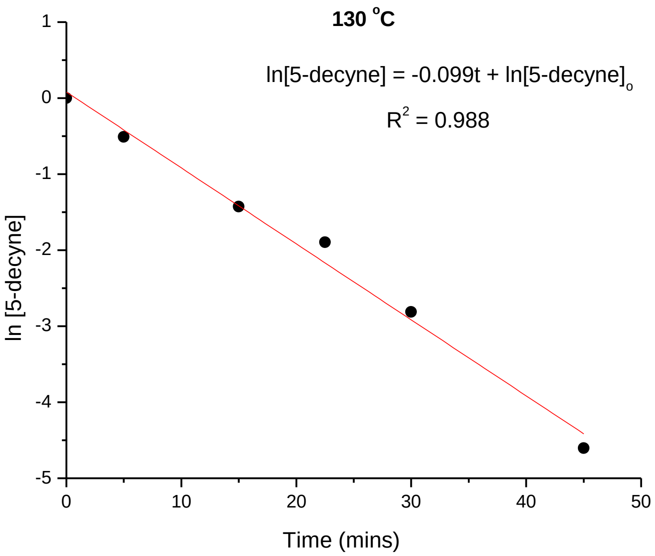

Time (min)	ln ([5-decyne])
0	0
5	−0.51
15	−1.43
23	−1.90
30	−2.81
45	−4.61

Figure S6. The linear plot of Pt catalyst **2A-**catalyzed hydrosilylations of 5-decyne (**4**) at 130 °C.

4. ^{1}H-NMR Spectra of Hydrosilylation Reaction Products

4.1. ^{1}H-NMR Spectrum of 5a and 5b

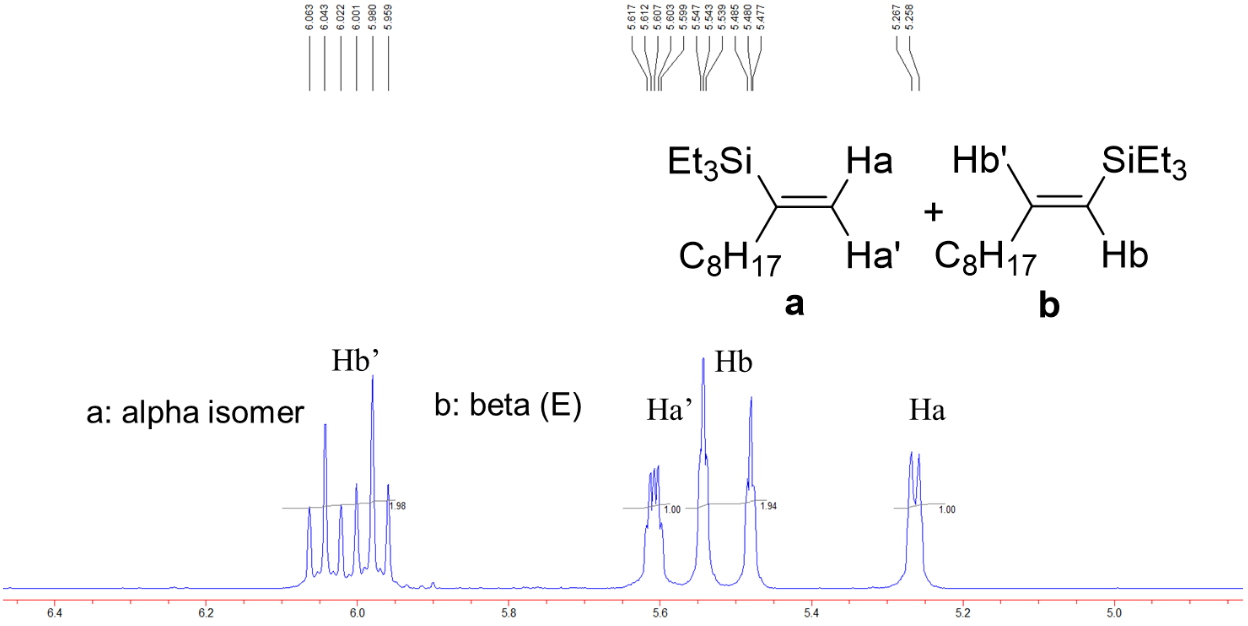

Figure S7. ^{1}H-NMR spectrum of hydrosilylation reaction of 1-decyne (**5**) and triethylsilane (**3**).

4.2. ^{1}H-NMR Spectrum of 6a and 6b

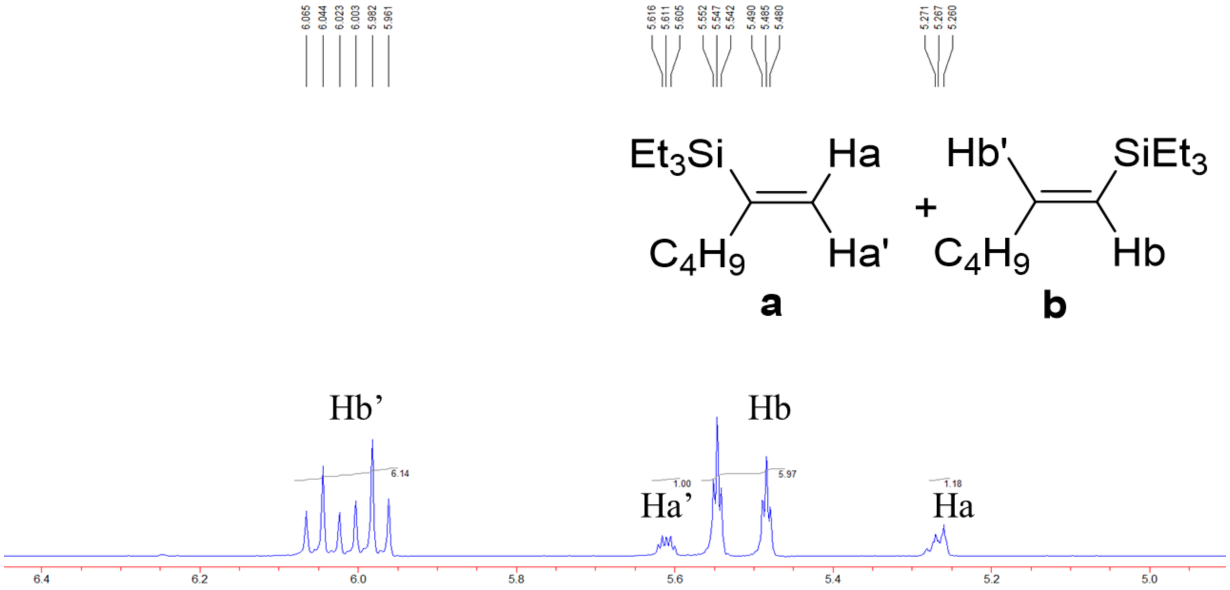

Figure S8. ^{1}H-NMR spectrum of hydrosilylation reaction of 1-hexyne (**6**) and triethylsilane (**3**).

4.3. ^{1}H-NMR Spectrum of 8a and 8b

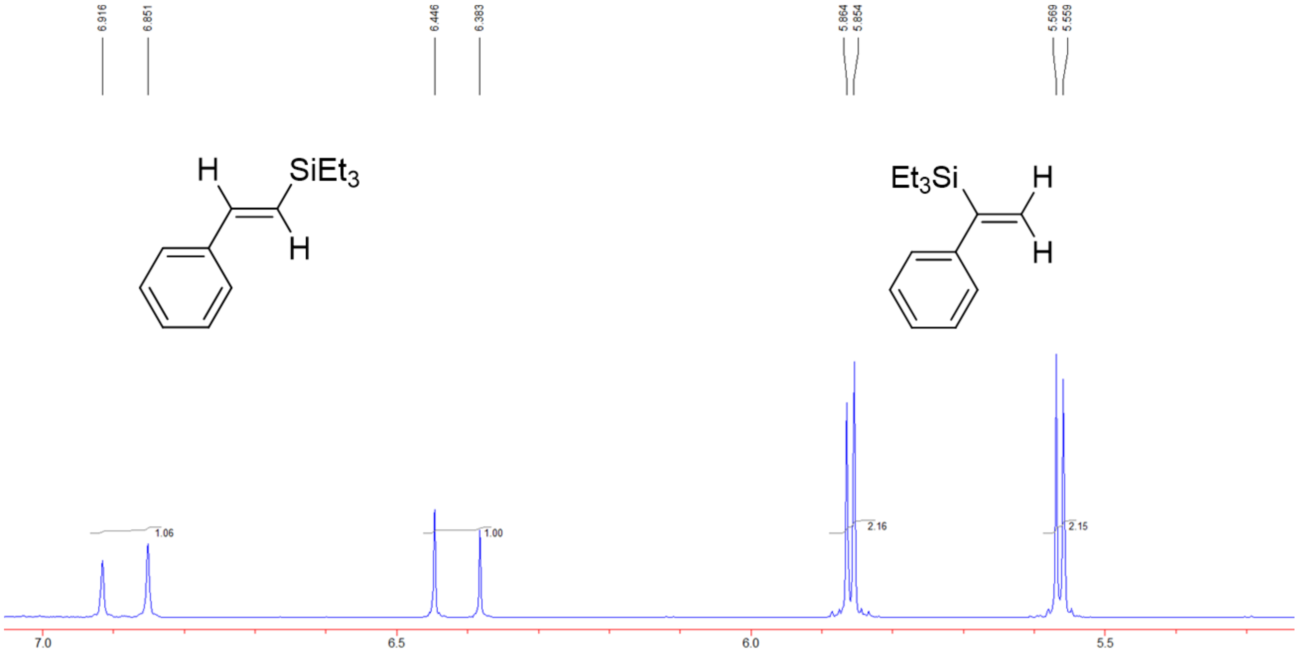

Figure S9. ^{1}H-NMR spectrum of hydrosilylation reaction of phenylacetylene (**8**) and triethylsilane (**3**).

5. Tables (S1–S3) from Recycling Studies

Table S1. Hydrosilylation reaction of 1-hexyne (**6**) and triethylsilane (**3**) under thermomorphic condition.

Cycle	Time (h)	6a:6b	Yield (%)[a]
**1**	0.5	1:4.66	>98
**2**	0.5	1:3.55	100
**3**	0.5	1:3.56	>99 (>91)
**4**	0.5	1:3.66	100
**5**	0.5	1:2.73	100
**6**	0.5	1:2.22	100
**7**	0.5	1:2.35	100 (92)
**8**	0.5	1:2.88	100 (>95)

Reaction conditions: T = 120 °C, cat. **2A** 1 mol%, **6** (164 mg, 2 mmol), **3** (232 mg, 2 mmol), solvent: 2 mL. a: Based on GC yield; isolated yields are given in parentheses.

Table S2. Hydrosilylation reaction of 4-fluorophenylacetylene **(9)** and triethylsilane (**3**) under thermomorphic condition.

Cycle	Time (h)	9a:9b	Yield (%)[a]
**1**	4	2.86:1	100
**2**	4	2.54:1	>99 (97)
**3**	4	2.58:1	>99 (>95)
**4**	4	2.21:1	100
**5**	4	2.30:1	100
**6**	4	2.58:1	100
**7**	4	2.47:1	100
**8**	4.5	2.05:1	100

Reaction conditions: T = 120 °C, cat. **2A** 1 mol%, **9** (240 mg, 2 mmol), **3** (232 mg, 2 mmol), solvent: 2 mL. a: Based on GC yield; isolated yields are given in parentheses.

Table S3. Hydrosilylation reaction of 4-methylphenylacetylene (**10)** and triethylsilane (**3**) under thermomorphic condition.

Cycle	Time (h)	10a:10b	Yield(%)[a]	TON
**1**	2	1.58:1	100 (95)	>99
**2**	2	2.10:1	>99	>99
**3**	2	2.13:1	99	>98
**4**	2	2.13:1	100 (>93)	>99
**5**	2	2.08:1	100	>99
**6**	2	2.08:1	100	>99
**7**	2	2.14:1	100	>99
**8**	4	1.98:1	100	>99

Reaction conditions: T = 120 ^{o}C, cat. **2A** 1 mol%, **10** (232 mg, 2 mmol), **3** (232 mg, 2 mmol), solvent: 2 mL. a: Based on GC yield; isolated yields are given in parentheses.

6. Computational Method

Theoretical calculations were performed using the Gaussian 09 packages. The crystal geometry of **55-8FCl-PtCl$_2$** (**2A**) was optimized using the B3LYP functional with a basis set of standard 6–31 G(d) for C, H, N, O, and F atoms and LANL2DZ for Pt and Cl atoms (Figure S10). Harmonic vibrational frequency analysis of the corresponding single molecule was carried out to make sure that the optimized structures represented the local energy minima.

6.1. Simulated structure of **55-8FCl-PtCl$_2$** (**2A**)

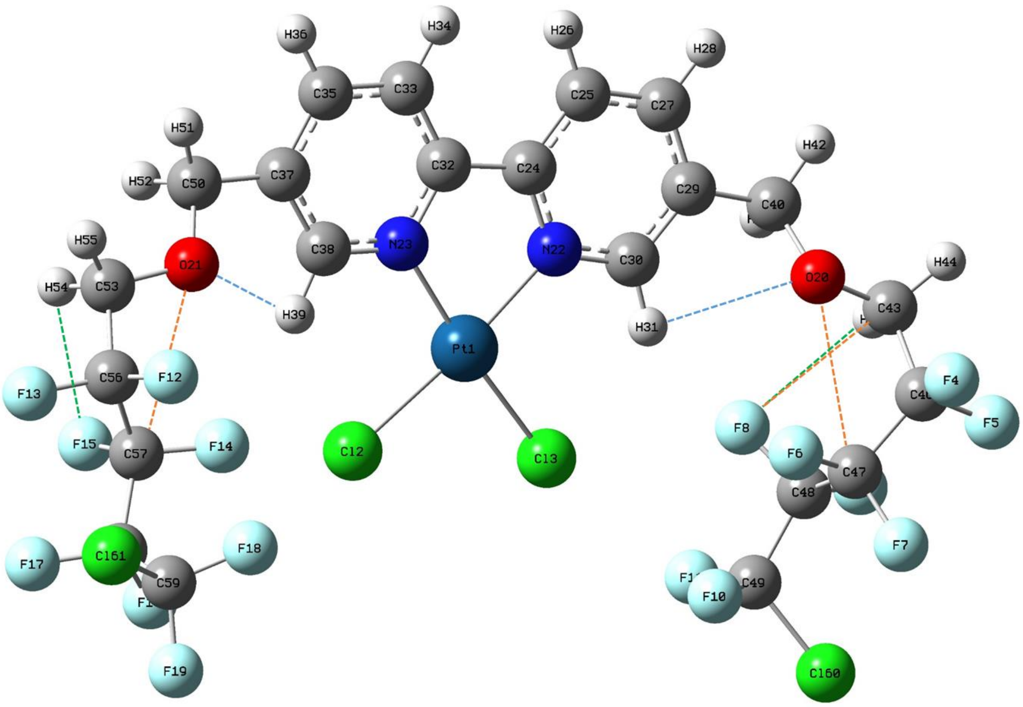

Figure S10. Theoretically optimized crystal geometry of **55-8FCl-PtCl$_2$** (**2A**). [The reverse U-shaped (with the metal halide, $PtCl_2$, inside)] [Note: from our recent studies on halogen bond, XB, it is possible that the O atom from each of CH_2OCH_2 spacer of two fluorous chains forms tetrel bond weak interactions with carbons from the two fluorous chains.] **Each O forms the intramolecular 5 membered ring with the pyridyl H (shown as the blue dash line)**. Each O atom also forms the tetrel bond (**TB**) with the C atom about three bonds away. The r(O21···C57) (left side) and r(O28···C47) (right side) are 2.99 and 3.00 Å, respectively. The distance (indicated by the two orange dash lines) of either O···C tetrel bond (TB) is significantly shorter than sum

(3.31 Å) of their vdw radii.] Additionally, there are other weak interactions which include TB and H-bonding in the system. For example, tetrel bonds (C43···F8) present between the methylene carbons (or H) and F atoms (4 bonds away).

6.2. Structure of **55-8FH-PtI₂** (showing the two ponytails to align themselves almost at the metal plane and to orient themselves at the both sides of the metal center)

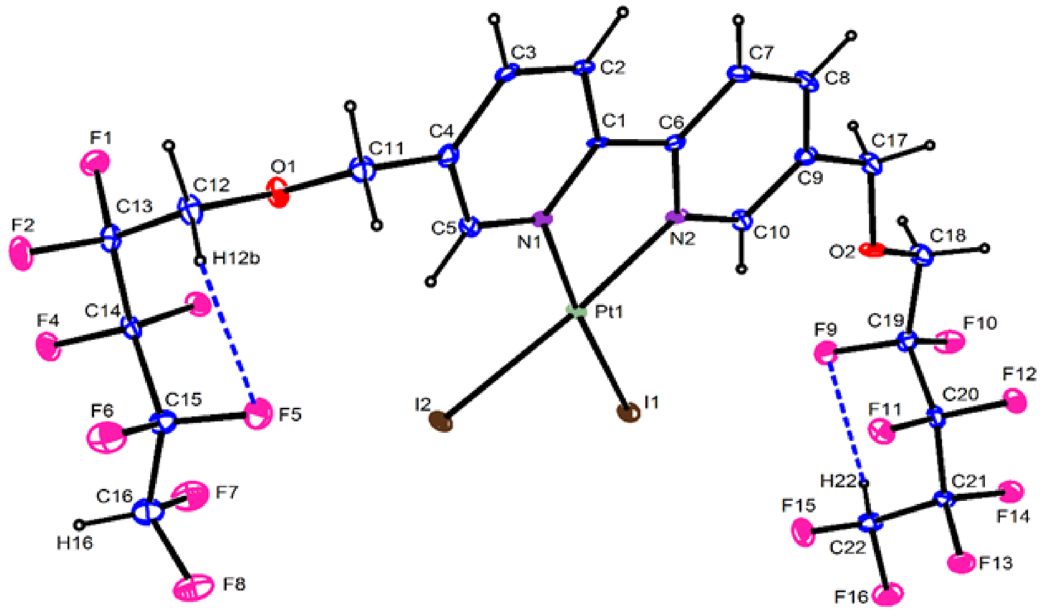

Figure S11. The structure of **55-8FH-PtI₂** which has the structure similar to the optimized structure of Pt complex (**2A**).[4] (which is shown as Fig. S10)

6.3. Structure of **55-8FH-PtI₂** (also showing the two ponytails to align themselves almost at the metal plane and to orient themselves at the both sides of the metal center)

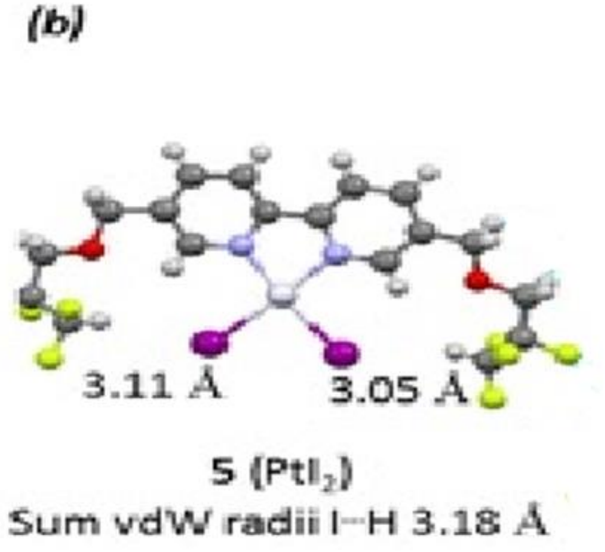

Figure S12. The structure of **55-4FH-PtI$_2$** which also has the reversed U-shaped structure contains the PtI$_2$ core within the U-shape.[5] [Figure S12 is taken from Figure S7b of the ref.5.] [Note: Pt: silver, I: purple, C: grey, O: red, N: nitrogen, H: white, F: greenish.]

7. Probes of a Series of Fluorinated Pt Complexes

7.1. Solubilities 55-3F-$PtCl_2$ in Bu_2O and DMF

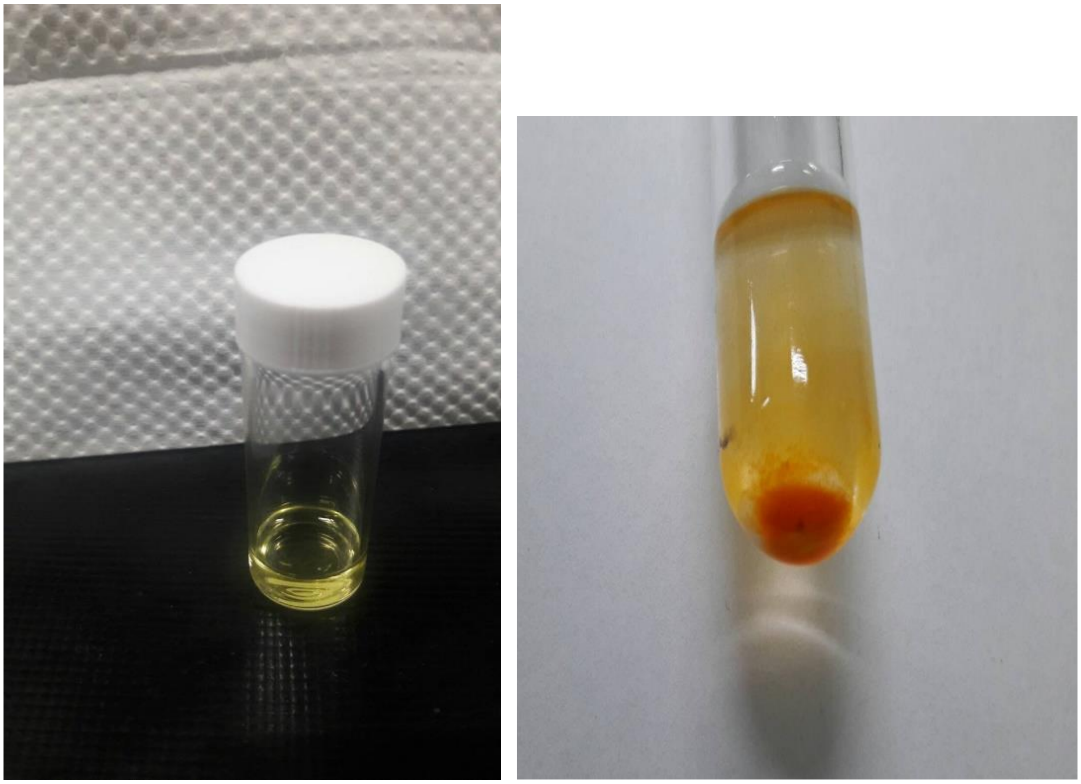

(a) (b)

Figure S13. (**a**). **55-3F-$PtCl_2$** is soluble in DMF at room temperature (RT). (**b**). **55-3F-$PtCl_2$** is moderately soluble in Bu_2O at RT.

55-3F-$PtCl_2$ is soluble in DMF at room temperature (RT). The image is shown in Figure S13a. It is moderately soluble in Bu_2O at RT (shown in Figure S13b) , so **55-3F-$PtCl_2$** does not meet the requirement of catalyst recovery at room temperature.

7.2. Solubility and Reactivities of 55-16FH-PtCl$_2$

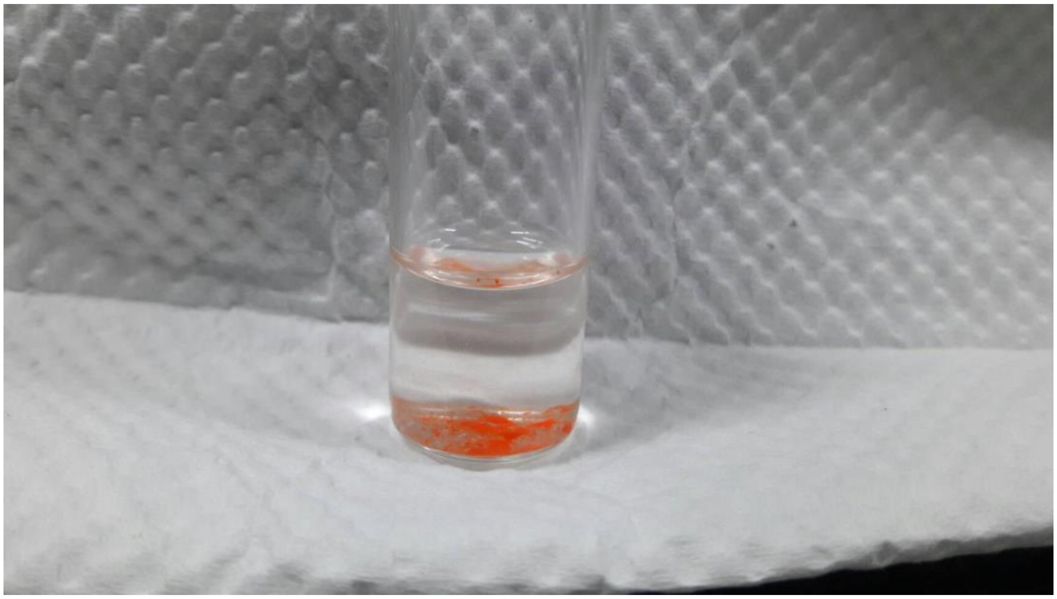

Figure S14. **55-16FH-PtCl$_2$ (3D)** is hardly soluble in Bu_2O at room temperature (RT).

Briefly, reactivity descriptions: The **55-16FH-PtCl$_2$**-catalyzed hydrosilylation reactions of 5-decyne in Bu_2O have been repeated 8 times with yieldings showing in Table S4 at 120 °C for 3 hours.

Table S4. Results of **55-16FH-PtCl$_2$** catalyzed hydrosilylation of 5-decyne with $HSiEt_3$ at 120 °C under thermomorphic mode.

Cycle	Time (h)	Yield (%)[a]
**1**	3	100
**2**	3	100
**3**	3	99
**4**	3	96
**5**	3	98
**6**	3	98
**7**	3	97
**8**	3	97

Reaction conditions: T = 120 °C; cat. **55-16FH-PtCl$_2$** 1 mol%, **4** (276 mg, 2 mmol), **3** (232 mg, 2 mmol), solvent: 2 mL. a: Based on GC yield.

8. Recycling Scheme

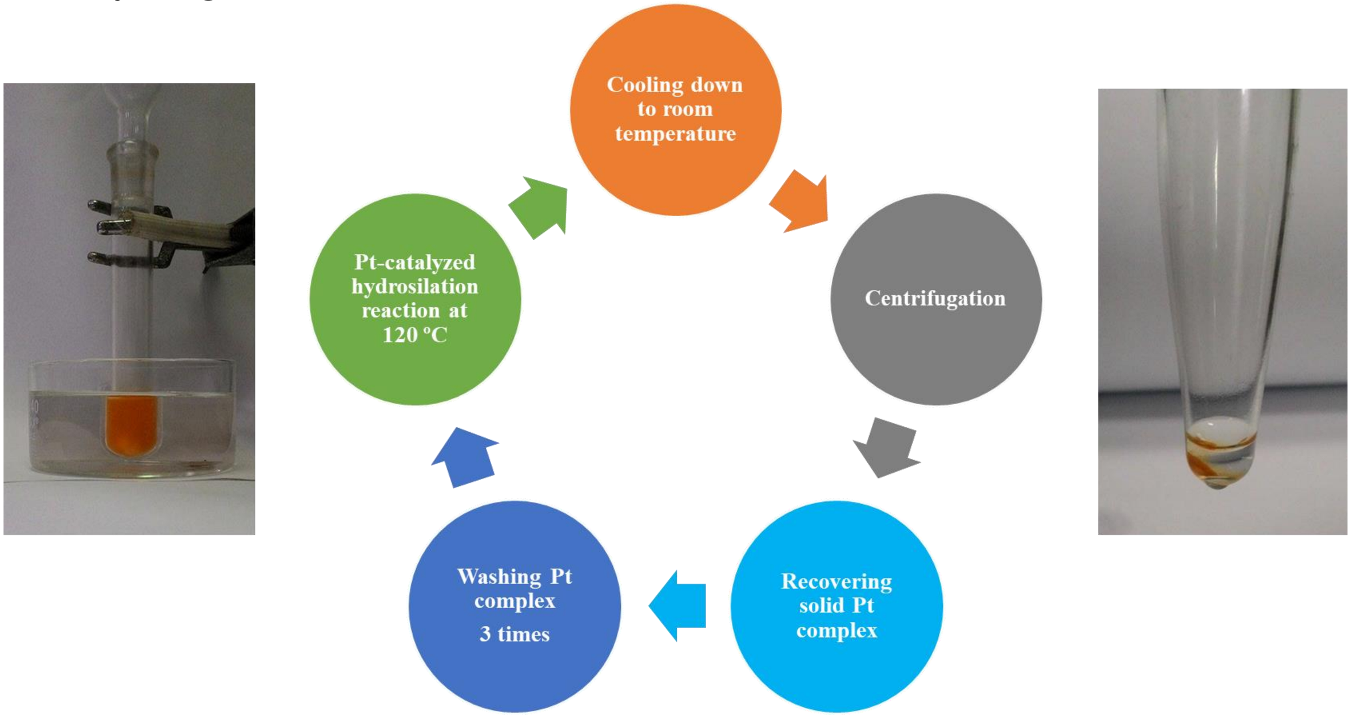

Scheme S1. The recycling procedure of the complex **2A** during the recoverable **2A**-catalyzed hydrosilylation[6–8].

References

1. Lu, N.; Ou, Y.-M.; Feng, T.-Y.; Cheng, W.-J.; Tu, W.-H.; Su, H.-C.; Wang, X.; Liu, L.; Hennek, M.D.; Sayler, T.S. Synthesis and characterization of polyfluorinated 2, 2′-bipyridines and their palladium and platinum complexes,[MX_2(bis ($R_fCH_2OCH_2$)-2,2′-bpy)](X = Cl, Br). *J. Fluor. Chem.* **2012**, *137*, 54–63.
2. Lu, N.; Chen, S.-C.; Chen, T.-C.; Liu, L.-K. Palladium-catalyzed Heck reaction under thermomorphic mode. *Tetrahedron Lett.* **2008**, *49*, 371–375.
3. Li, C.-K.; Ghalwadkar, A.; Lu, N. Recoverable cationic Pd-catalyzed Heck reaction under thermomorphic mode. *J. Organomet. Chem.* **2011**, *696*, 3637–3642.
4. Lu, N.; Zheng, J.H.; Lin, L.C.; Liu, L.K.; Chiang, H.F.; Li, T.Y.; Wen, Y.S.; Yang, C.K.; Chen, S.W.; Thrasher, J.S. Studies of two different types of intramolecular C–H···F–C interactions from polyfluorinated diiodometal (II) diimine complexes. *J. Chin. Chem. Soc.* **2019**, *66*, 31–40.
5. Lu, N.; Lu, Y.J.; Huang, J.Y.; Chiang, H.F.; Kung, C.C.; Wen, Y.S.; Liu, L.K. The Evolution Aspect in the Crystallization Process of [5,5′-($HCF_2CF_2CH_2OCH_2$) 2-2,2′-bpy] MX_2 (M= Pt, Pd; X = I, Br): Role of the Intramolecular CF_2—H…X (—M) Hydrogen Bonds. *J. Chin. Chem. Soc.* **2018**, *65*, 613–627.
6. Lu, N.; Chung, W.-C.; Chiang, H.-F.; Fang, Y.-C.; Liu, L.-K. Recoverable platinum bis (fluoro-ponytailed) bipyridine complex as catalyst for hydrosilylation of alkynes under thermomorphic condition. *Tetrahedron* **2016**, *72*, 8508–8515.
7. Mollar Cuni, A.; Borja, M.P.; Guisado-Barrios, G.; Mata Martínez, J.A. A Platinum molecular complex immobilised on the surface of graphene as active catalyst in alkyne hydrosilylation. **2020,** *45*, 4254–4262.
8. Meister, T.K.; Riener, K.; Gigler, P.; Stohrer, J.r.; Herrmann, W.A.; Kühn, F.E. Platinum Catalysis Revisited: Unraveling Principles of Catalytic Olefin Hydrosilylation. *ACS Catalysis* **2016**, *6*, 1274–1284.
